# Soil pH, Calcium Content and Bacteria as Major Factors Responsible for the Distribution of the Known Fraction of the DNA Bacteriophage Populations in Soils of Luxembourg

**DOI:** 10.3390/microorganisms10071458

**Published:** 2022-07-19

**Authors:** Perrine Florent, Henry-Michel Cauchie, Malte Herold, Stéphan Jacquet, Leslie Ogorzaly

**Affiliations:** 1 Environmental Research and Innovation Department (ERIN), Luxembourg Institute of Science and Technology (LIST), 4422 Belvaux, Luxembourg; perrine.florent@list.lu (P.F.); henry-michel.cauchie@list.lu (H.-M.C.); malte.herold@list.lu (M.H.); 2 Faculté des Sciences, de la Technologie et de la Communication (FSTC), Doctoral School in Science and Engineering (DSSE), University of Luxembourg, 4365 Esch-sur-Alzette, Luxembourg; 3 INRAE, UMR CARRTEL, Université Savoie Mont Blanc, 74200 Thonon-les-Bains, France; stephan.jacquet@inrae.fr

**Keywords:** DNA bacteriophages, soil, metagenomics, bacteria, soil properties

## Abstract

Bacteriophages participate in soil life by influencing bacterial community structure and function, biogeochemical cycling and horizontal gene transfer. Despite their great abundance, diversity, and importance in microbial processes, they remain little explored in environmental studies. The influence of abiotic factors on the persistence of bacteriophages is now recognized; however, it has been mainly studied under experimental conditions. This study aimed to determine whether the abiotic factors well-known to influence bacteriophage persistence also control the natural distribution of the known DNA bacteriophage populations. To this end, soil from eight study sites including forests and grasslands located in the Attert River basin (Grand Duchy of Luxembourg) were sampled, covering different soil and land cover characteristics. Shotgun metagenomics, reference-based bioinformatics and statistical analyses allowed characterising the diversity of known DNA bacteriophage and bacterial communities. After combining soil properties with the identified DNA bacteriophage populations, our *in-situ* study highlighted the influence of pH and calcium cations on the diversity of the known fraction of the soil DNA bacteriophages. More interestingly, significant relationships were established between bacteriophage and bacterial populations. This study provides new insights into the importance of abiotic and biotic factors in the distribution of DNA bacteriophages and the natural ecology of terrestrial bacteriophages.

## 1. Introduction

Soil is a complex compartment where biodiversity and interactions between microbes, animals and plants contribute to its life and optimal functioning. Generally, plants provide organic matter as well as nutrients to soil (e.g., nitrogen, carbon, phosphorous) and are responsible for its structure through the development of the rhizosphere [1,2]. Soil-dwelling animals help to degrade and mix organic matter throughout the soil and participate in the recycling of nutrients from dead biomass [3,4]. Microbes, including fungi, archaea and bacteria, play a key role in organic matter decomposition, element cycling and nutrient availability to plants [5,6,7]. This microbial activity, particularly due to the quantity, diversity and functional roles of prokaryotes, is considered central to soil processes [8,9,10]. Viruses are also biological entities fully part of soil biodiversity with essential but relatively unexplored functions [11].

Since the beginning of research on viral diversity in soils in the 1970s [11,12], knowledge of soil viruses has been expanded well, especially for bacteriophages (bacteria-infecting viruses), whose abundance can reach up to 10^10^ particles per gram of soil [13,14]. Bacteriophages are ubiquitous in the environment and can be found in agricultural soils, forest soils (approximately 10^9^ bacteriophages per gram of dry soil), wetlands, or even cold deserts (approximately 10^3^ bacteriophages per gram of dry soil) [13]. Bacteriophages seem to be more abundant in wetlands than in forests due to high soil water content, but less abundant in agricultural soils than in forests where the soil temperature is lower [13]. In fact, many abiotic factors may be involved in the distribution, abundance and diversity of bacteriophages in soils. However, most of them have only been explored to investigate their effect on bacteriophage persistence. Temperature is recognised as an important, if not the main, factor in viral inactivation [15]. The ideal temperature range to prevent viral inactivation is considered to be 4 to 10 °C, where bacteriophages are generally stable at pH values between 5 and 9, but can be degraded under strongly alkaline (pH > 11) or acidic (pH < 2) conditions [16,17,18]. These extreme pH conditions can inactivate virus particles through the denaturation of their proteins or nucleic acids [18,19,20,21]. In addition, soil texture, especially clay type, alters the detection of bacteriophage as it strongly influences adsorption and retention phenomena in the soil. Indeed, clay soils present a very dense structure with a low permeability and a large specific surface area, which is auspicious for higher adsorption–desorption reactions with bacteriophages [22]. In addition, due to hydrophobicity interactions and the presence of electrical charges, bacteriophages tend to adsorb more onto clay surfaces than onto sandy or silty soil particles [23]. This process also helps to protect bacteriophages from other inactivating factors [11,24].

However, knowledge of the influence of these abiotic factors on bacteriophage persistence has been mainly gained under experimental conditions using bacteriophage models (e.g., ΦX174, MS2), which may not be transferable when studying environmental samples [19,21,25,26]. Indeed, the natural bacteriophage diversity and the impact of such abiotic factors remain largely underexplored in terrestrial compared to aquatic environments [27,28,29]. Regarding the studies exploring the diversity of bacteriophages on in situ terrestrial ecosystems, virus-like particles (VLPs) or viral operational taxonomic units (vOTUs) have dominantly been studied [30,31,32,33]. While the former does not allow the identification of viruses, the latter is rather a proxy for viral species. Therefore, the use of metagenomics in determining the taxonomical classification of viruses and their respective diversity would not only allow completing the knowledge about the influence of abiotic factors on the bacteriophage species communities but also explore the relationship with the bacterial communities.

Coevolution between bacteriophages and their bacterial hosts is widely recognised, with reciprocal influences [11,34]. Bacteriophages are unable to self-replicate and therefore must infect one or more bacterial hosts to replicate and spread. As agents of bacterial mortality, bacteriophages are likely to have a significant influence on bacterial community dynamics, structure and function, as well as biogeochemical cycling, horizontal gene transfer processes and the pre-decomposition of the organic matter [35,36]. However, viral-mediated mortality induces interest at the expense of understanding the influence of bacteria on bacteriophage occurrence in soils [37,38,39].

With the advent of viral metagenomics, databases increasingly include new viral sequences, which participate in the rapid expansion of our understanding of the virosphere. Based on the viruses already identified, the influence of selected abiotic factors (i.e., soil properties, vegetation and land use) on the distribution of the known DNA bacteriophages can be assessed in different types of soil. Therefore, in this study, we used a combination of approaches and methods (i.e., physicochemical analysis, metagenomics, reference-based bioinformatics, direct counting and statistics) to obtain an original picture of the natural viral and bacterial diversities and their ecological interactions with soil properties in terrestrial environments of Luxembourg. The aim of the study was to determine whether the abiotic factors well known to influence bacteriophage persistence also control the natural distribution of the known DNA bacteriophage populations in the studied soil samples, and to analyse the role of bacterial populations in this phenomenon. Two main hypotheses were tested: (i) abiotic factors (i.e., pH, cations) well known to affect bacteriophage survival also have an impact on their diversity and distribution in terrestrial environments, and (ii) the bacterial community present in the soil influences the relationship between bacteriophages and soil properties.

## 2. Materials and Methods

### 2.1. Studied Area

**Figure 1:**
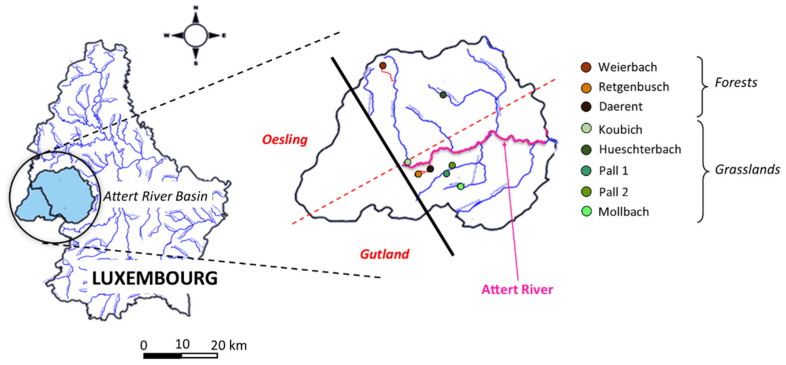
Map of the Attert River basin with the location of the eight sampling sites (modified from Geoportail.lu). Brown colours: forest sites; green colours: grasslands. With an oversimplified scheme, the plain black line represents the border between the Luxembourg and Belgian sides of the basin while the dashed red line is the limit between Oesling and Gutland regions. The Attert River is highlighted in pink, the affluents of the Attert River are in blue and the affluents of affluents, in orange.

The Attert River basin area reaches 310 km^2^ and covers a wide range of bedrock geologies and land use [40], ultimately being representative of Luxembourg’s main physiographic characteristics. Its northern boundary lies on schist bedrock (belonging to the Ardennes massif; Oesling region), while its southern part exhibits alternating sedimentary layers of marls and sandstone (representative of the eastern limit of the Paris basin; Gutland region). The Attert River originates near Nobressart (Belgium) and extends over 38 km from west to east, joining the Alzette River basin in Bissen, Luxembourg. The study is focused on the Luxembourg side of the Attert River basin (Figure 1, Table 1) where 8 sub-basins have been selected compiling 3 forested sites and 5 grassland sites. All of them are located near one of the tributaries of the Attert River.

The first forest site is the Weierbach sub-basin located in the northwest of the Attert River basin, in the Oesling region. The two other forest sub-basins are Retgenbusch and Daerent, located on the same tributary of the Attert River basin in the southwestern part of the river (Gutland region). All forest sites have distinct vegetation types; Weierbach is a beech forest, while Daerent is an oak forest and Retgenbusch is a spruce forest.

The first grassland site, Hueschterbach, is the only sub-basin located in the Oesling region and is characterised by schist bedrock. The four other grassland sites are in the Gutland region: Mollbach a sub-basin located in the southwest of the Attert River basin; Pall located in the north of the Mollbach sub-basin, for which two distinct sites (Pall 1 and Pall 2) were chosen on the tributary of the same name; the sub-basin Koulbich located in the northwest of the Attert River basin is known for pasture activities of barley and wheat cultures.

### 2.2. Sample Collection

The field campaign was conducted on 12 September 2019. The topsoil was collected for all selected sites cited above. Five to seven replicates were randomly sampled within a delimited surface of 25 m^2^, with a soil auger (3 cm in diameter) on the first 20 cm of depth. To exclude the intra-site spatial variability and to ease laborious shotgun metagenomic and bioinformatic analyses, 5 to 7 soil replicates were pooled altogether to set up one sample per site, representing around 300 g per site [41,42,43]. All soil samples were split into two parts, either for physicochemical analyses or stored at −20 °C for molecular analyses.

### 2.3. Soil Characterisation

The characterisation of the soil properties includes first the pH measurements made on 10 g of soil according to the ISO 10,390 [44] for pH_H2O_ and pH_KCl_ and to VDLUFA A5.1.1 [45] for pH_CaCl2_. Then, the nutrients dosing of phosphorus (P_2_O_5_) and potassium (K_2_O) was performed according to the VDLUFA A6.2.1.1 [46] using 5 g of soil with 100 mL of Ca-acetate-lactate at pH = 4.1. On the other hand, the dosing of magnesium (Mg) and sodium (Na) was conducted following the VDLUFA A6.2.1.7 [47], using 10 g of soil with 100 mL of CaCl_2_ (0.01 M). In addition, the carbon-to-nitrogen ratio (C/N) was calculated after quantifying the total organic carbon (C_org_) and the total nitrogen (N_tot_) both on 300 g of soil, according to ISO 10,694 [48] and ISO 13,878 [49], respectively. The cation exchangeable capacity (CEC) and cation saturation rate (S/T) were analysed according to the ISO 23,470 [50] on 2.5 g of soil. From the extract CoHex, calcium (Ca^2+^), magnesium (Mg^2+^), potassium (K^+^) and sodium (Na^+^) CoHex were analysed by ICP-OES (inductively coupled plasma–optical emission spectrometry). Finally, the soil granulometry was determined by a sieving method on 10 g of soil mixed with 100 mL of H_2_O_2_ and 10 mL HCl [51].

### 2.4. Viral Quantification

#### 2.4.1. Viral Elution and Concentration

After the optimisation of the elution and concentration protocol (Supplementary Materials, Section S1), 5 g of soil, previously stored at −20 °C, was placed at room temperature and then mixed with 15 mL of the eluent solution, i.e., 3% beef extract and 0.05 M glycine (at pH = 9.5) and shaken vigorously using an orbital shaker for 30 min at 250 rpm (rotation per minute) at 4 °C. The samples were then centrifuged (Eppendorf 5801R) at 5000× *g* for 15 min on a fixed rotor at 4 °C. The supernatant was collected and underwent a 0.22 µm Millex^®^ sterile filter unit with a Millipore Express^®^ PES membrane (SLGP033RS, Merck Millipore Ltd., Tullagreen, Ireland), followed by ultracentrifugal filtration using an Amicon Ultra-15 50 kD (UFC905024, Merck, Darmstadt, Germany), upon the recommendations of the manufacturer. After centrifugation at 5000× *g* for 15 min on a fixed rotor at 4 °C, the concentrate solutions, with a volume varying from 0.3 to 3 mL depending on the site, were finally transferred into 2 mL cryotubes and stored at 4 °C.

#### 2.4.2. Viral-like Particles (VLPs) Counting

From the viral concentrates obtained after the viral elution and concentration steps, the abundances of VLPs were determined by flow cytometry (i.e., a benchtop FACSCalibur), after the samples were divided into 3 technical replicates. Briefly, after homogenisation by centrifugation, the viral sample was fixed using glutaraldehyde (0.25% final concentration) and mixed with autoclaved and <0.02 µm filtered TE buffer (0.1 mM Tris and 1 mM EDTA, pH 8), SYBR© Green I (at a final concentration of 5 × 10^−5^, Molecular Probes, Eugene, OR, USA). The samples underwent a 10 min heat treatment at 70 °C before the Flow CytoMetry (FCM) analysis [52].

### 2.5. Identification of the Microbial Populations

#### 2.5.1. Total DNA Extraction and Shotgun Metagenomics Sequencing

After a comparison of different protocols (Supplementary Materials, Section S2), DNA extraction was performed using the DNeasy^®^ PowerSoil^®^ Kit (Qiagen, Hilden, Germany) from 0.25 g of frozen soil (−20 °C) for each site, according to the manufacturer’s recommendations The DNA elution step was performed in 60 μL instead of 100 µL to increase the DNA concentration. The final DNA solutions were stored at −20 °C until further analyses.

From the total extracted DNA, library preparations were completed using the Nextera DNA Flex Kit (Illumina, San Diego, CA, USA) according to the manufacturer’s instructions. Paired-end sequence reads were generated using the Illumina NovaSeq 600 (2 × 150 bp).

#### 2.5.2. Reference-Based Bioinformatics

The raw sequencing reads were quality controlled and Illumina adapters were trimmed with Trimmomatic 0.39 [53]. Assemblies were performed separately for each sample using SPAdes v3.15.4 [54] with the “meta” option and default parameters Taxonomic classification of contigs was carried out against the complete non-redundant (nr) microbial protein sequence database (released version of April 2022) from NCBI using MMseqs2 v13.45111 [55] and the easy-taxonomy workflow that determines ancestors by searching translated ORFs (translation Table 11) [56]. Quality-filtered reads from each metagenome were mapped back to the assembled contigs using minimap2 v2.24 with the “sr” preset [57] and sorted and indexed with SAMtools v1.15.1 [58]. Finally, metagenomic profiles were exported as comma-separated value (CSV) files. Metagenomic abundances were calculated based on the number of reads mapping to each contig. Mapped reads were divided by contig length and the total number of mapped reads per sample [59,60].

### 2.6. Statistical Analyses

Statistical analyses were performed with RStudio, version 1.4.1106, and R 4.0.4 [61]. Figures were generated using the package ‘ggplot2’. Regarding the ecological diversity, both α and β-diversities of bacteria and DNA bacteriophages were studied. The α-diversity is the average number of distinct species groups at a local scale, considering the richness of a population [62]. As popular α-diversity indexes, the Shannon index (H) and the species richness (S) were calculated for each site, using the R package ‘vegan’ [63]. Then, the evenness (E), which defines the distribution of the abundance of the species in a community, was calculated as follows [64,65]:

$$E=\frac{H}{\log\left(S\right)}$$

The β-diversity is all changes in the species composition of a population between several habitats in a region. This diversity considers both the richness and evenness of populations [66]. Using Bray–Curtis dissimilarities, a multivariate method, the RDA (redundancy analysis), was performed to compare the different sites as a function of the bacteriophage populations at the genus level, through the analysis of their variation from one another. To build the RDA, a selection of the 10 most abundant genera was first performed, as rare genera might generate noise in the analysis). Then, abiotic factors (i.e., sand, clay, silt content, soil pH, magnesium concentration, carbon-to-nitrogen ratio and Ca^2+^ concentration) were selected by removing the variables for which strong correlations (>r = 0.80) were found to avoid co-variations, using the function ‘pairs’. In addition, all abiotic factors that significantly constructed the RDA were kept (i.e., pH and Ca^2+^). The determination of the explanatory variables for the RDA was conducted using ‘vegan’ and correlations of the abiotic factors with each axis were defined using the package ‘psych’. Permutational multivariate analysis of variance (PERMANOVA) was conducted to establish the impact of some abiotic factors on the distribution of the DNA bacteriophages. From the calculation of the Bray–Curtis dissimilarities to the PERMANOVA and RDA, the package ‘vegan’ was used to conduct these analyses. Finally, a co-correspondence analysis (CoCA) was performed to assess the co-variation of the distribution of the total bacterial and bacteriophage genera obtained from the metagenomic analyses, among the different sites. This analysis is based on contingency table data and highlights co-similarities between two groups of variables (here, bacterial species data and DNA bacteriophage genera data). CoCA was performed using the package ‘coca’, combined with a Mantel test using Pearson correlations.

## 3. Results

### 3.1. Soil Description

The granulometric analysis of the soil revealed similar textures between the sites (Table 2). Indeed, selected sites are mainly silty, especially Mollbach and Retgenbusch, for which the silt content is around 52–58%. However, Weierbach and Pall 1 are clay loam with approximately 42% silt, while Daerent and Huechterbach are sandy loam with 53% sand. Concerning Pall 2, the proportion for the different textures are rather equal (28% clay, 38% silt, 34% sand). Koulbich is a loam soil, with a similar proportion of silt and sand (≈40%) but little clay (19%). The gravimetric water content (GWC) was the highest for Weierbach (24.6%) and Pall 1 (20.9%) and the lowest for Koulbich (10.8%).

The physical-chemical properties of soils are reported in Table 2. Within the forest sites, the soils of Weierbach and Retgenbusch are highly acidic (respectively, pH = 4.1 and 4.5) and their respective cation exchange capacity and calcium concentration are quite low (CEC of Retgenbusch = 2.6 milliequivalent per 100 g (meq per 100 g) and Ca^2+^ = 1.6 meq per 100 g, CEC of Weierbach = 8.5 meq per 100 g and Ca^2+^ = 2.1 meq per 100 g). Their nutrient content was also relatively poor, especially for phosphorus (P_2_O_5_) and potassium (K_2_O), suggesting that these soils are not very fertile. In addition, the cation saturation rate (S/T) of Retgenbusch is superior to 85% (S/T = 101%), which can explain the high calcium content in the soil, while the soil of Weierbach shows a regular calcium intake (20% ≤ S/T = 42% ≤ 85%). The soil of Weierbach is distinguished by a slow organic matter decomposition (carbon-to-nitrogen ratio or C/N = 18 and high C_org_ content), displayed by a need for nitrogen to ensure an efficient organic decomposition. The soil of Daerent is fertile (CEC = 17.1 meq per 100 g, pH = 6.3, K_2_O = 21 mg per 100 g of dry soil) and has a maximum decomposition rate (C/N = 10). Regarding the grassland sites, Pall 1, Pall 2 and Mollbach soils are all fertile (CEC = 23.7, 17.2, 14.3 meq per 100 g, respectively) with a high concentration of calcium (Ca^2+^ = 16.6, 15.1 and 13.1 meq per 100 g), while Koulbich and Hueschterbach are not quite (CEC = 7.4 and 8.5 meq/100 g, respectively) with lower calcium concentrations (Ca^2+^ = 5.8 and 8.7 meq per 100 g). However, all of them are saturated in calcium, with a cation saturation rate superior to 100%. Finally, the grassland soils have a rapid decomposition (C/N ≈ 8.5) with a maximum organic decomposition for Pall 1 (C/N = 9.8) and have a favourable pH (pH ≈ 6–6.5) and mineral nutrient for plant growth.

### 3.2. Description of the DNA-Bacteriophage Populations

#### 3.2.1. VLPs Enumeration

**Figure 2:**
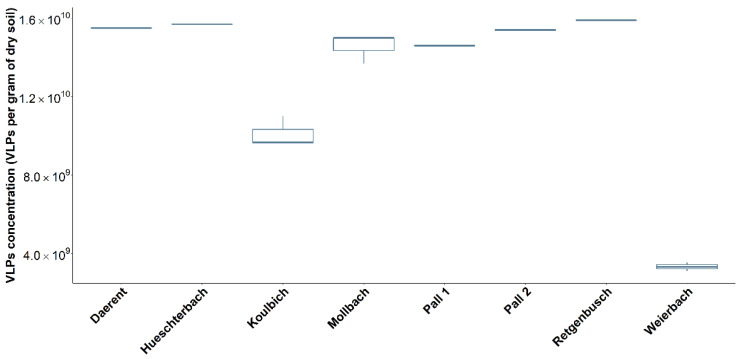
Boxplot of the virus-like particles (VLPs) concentration per gram of dry soil for all studied sites.

The average concentration of VLPs found in every soil was about 10^10^ VLPs per gram of dry soil (Figure 2). The highest concentrations were recorded for the soils of Retgenbusch, Hueschterbach, Daerent and Pall 2 with approximately 1.5 × 10^10^ VLPs per gram of dry soil. The lowest concentration was for the soil of Weierbach with 3.3 × 10^9^ VLPs per gram of dry soil. and displayed a 0.48 log difference with the lowest VLPs concentration (after the Weierbach), Koulbich and 0.67 log with Hueschterbach, the highest VLPs concentration. Finally, Koulbich showed on average a lower concentration of 0.18 log compared to all the other sites.

#### 3.2.2. Taxonomic Classification

The statistics of the shotgun metagenomic sequencing are reported in Table 3.

**Figure 3:**
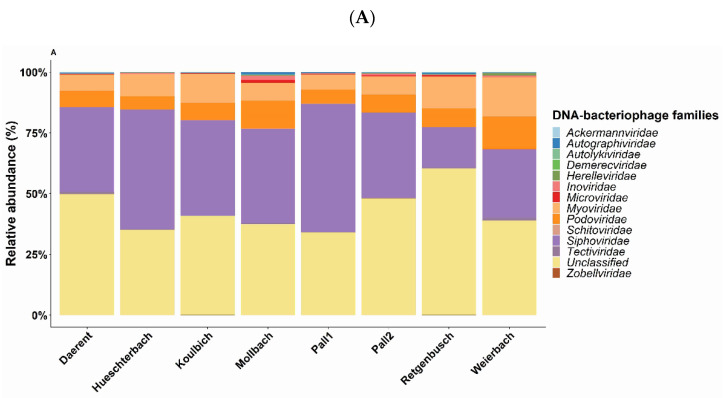

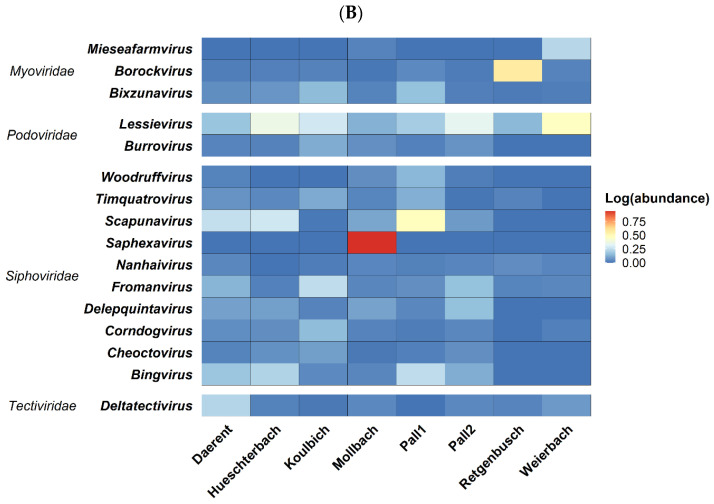
(**A**) Bar charts of the relative abundance (in %) of DNA bacteriophage families from the known fraction of all studied sites. (**B**) Heatmap of the most occurrent DNA bacteriophage genera identified (selection of genera with a log-abundance above 0.24) as a function of the sites. The gradient represents the log-transformed abundance, where red colour represents the most abundant genera and blue, the least ones.

The shotgun sequencing produced between 40 to 57 million paired reads per sample generating an average of 6.2 million contigs after assembly. From 75 to 99% of the total raw reads mapped back to the contigs. Of these mapped reads, between 0.01% and 0.04% were virus-affiliated reads (Supplementary Table S1), corresponding to a total of 37 known viral families for all sites included. Among these families, 13 DNA bacteriophage families were identified and represented between 89% and 96% of all virus-affiliated reads (Figure 3A). The DNA bacteriophage composition was strongly characterised by the *Caudovirales* order with between 75% and 93% of the total metagenomics identification. This high proportion was mainly due to the high abundance of *Siphoviridae* (13.9% to 49%), *Myoviridae* (5% to 12%) and *Podoviridae* (4% to 10%). *Caudovirales* gathers all the identified DNA bacteriophage families, except for Autolykiviridae (unclassified order) in Daerent and Mollbach soils, *Microviridae* (Petitvirales) and *Tectiviridae* (order Kalamavirales) both present in every site. By investigating each site, *Siphoviridae* was found to always be the most identified family, especially in the soils of Pall 1, Hueschterbach, Mollbach, Koulbich and Pall 2 (with, respectively, 49.58%, 44.83%, 35.15% and 34.80%) while its abundance was the lowest for Retgenbusch (13.9%). Regarding *Myoviridae* and *Podoviridae*, the former was highly abundant in Koulbich (10.65%), Retgenbusch (10.9%) and Weierbach (12.3%) while the latter was rather highly present in the soils of Weierbach (10.2%) and Mollbach (10.3%). It is noteworthy that most of the assigned DNA bacteriophages from the libraries belonged to the unclassified *Caudovirales* (between 29% and 51%). Of the two ssDNA bacteriophage families, i.e., *Inoviridae* and *Microviridae*, both were detected by the metagenomics with a low abundance in all sites (between 0.09% and 1.6% for the former and between 0.06% and 1% for the latter), while all the previously mentioned families are dsDNA bacteriophages.

To visualise the composition of the DNA bacteriophage population, a heatmap on the log-transformation abundances, at the genus level, was performed on the 10 most abundant genera, all sites included (Figure 3B). Genus *Lessievirus* (*Podoviridae*) was detected in every site, and more specifically in the soil of Weierbach, for which *Mieseafarmvirus* (*Myoviridae*) was also present. The soil of Mollbach was specifically represented by *Saphexavirus* (*Siphoviridae*), while in Retgenbusch, *Borockvirus* (*Myoviridae*) was the most abundant genus. Pall 1 was found to be mainly represented by *Scapunavirus* (*Siphoviridae*) followed by *Bingvirus* (*Siphoviridae*). *Scapunavirus* was also found in a slightly higher abundance in Daerent and Hueschterbach. Koulbich and Pall 2 did not display high abundances for specific genera, but still showed the presence of *Fromanvirus* (*Siphoviridae*) besides *Lessievirus*.

### 3.3. Impact of the Soil Properties on Known DNA-Bacteriophage Distribution

#### 3.3.1. α-Diversity: Known DNA-Bacteriophage Diversity within Sites

The DNA bacteriophage α-diversity of the known microbial fraction of each soil was investigated using the Shannon index, species evenness and species richness (Table 4). All sites displayed similar diversity (around H = 2) where Retgenbusch was the least diverse soil (H = 2.31) and Weierbach the most one (H = 2.83). Indeed, the soil of Weierbach presented the lowest number of DNA bacteriophage species (S = 91) and the highest evenness (E = 0.63). A one-way analysis of variance (one-way ANOVA) was performed on the Shannon index to analyse the hypothetical impact of the selected soil properties on the DNA bacteriophage diversity within the sites. As a result, none of the soil properties showed a significant effect on the DNA bacteriophage alpha-diversity.

#### 3.3.2. β-Diversity: Known DNA-Bacteriophage Diversity between Sites

The β-diversity analysis allowed comparing the DNA bacteriophage subset populations from one site to another. The PERMANOVA test (Table 5) performed on the DNA bacteriophage genera abundancy did not reveal a significant effect of the vegetation (i.e., forest and grasslands) on the distribution of the DNA bacteriophages (*p*-value = 0.075, α = 0.05).

**Figure 4:**
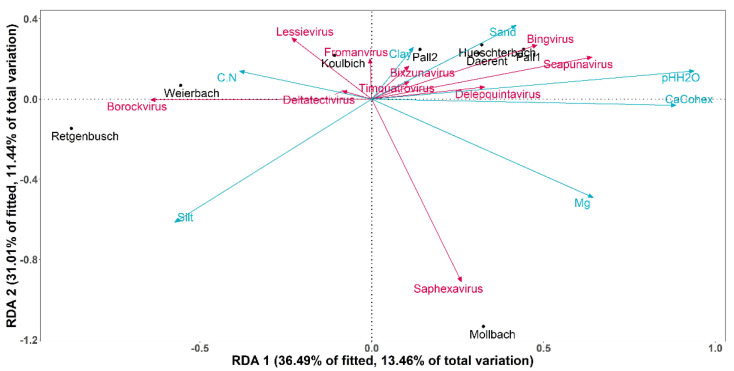
Biplot of redundancy analysis (RDA) on the 10 most abundant genera of DNA bacteriophage in relation to the soil properties of each site. The pink vectors represent the DNA bacteriophage genera with a Hellinger transformation, the blue vectors display the selected soil properties and the sites are shown in black. The RDA was built over the two first dimensions, with 36.49% of the fitted variations explained by RDA1 and 31.01% by RDA2. (C.N = carbon to nitrogen ratio; Mg = magnesium concentration; pHH_2_O = soil pH measured in water, CaCohex = calcium cation available).

However, soil pH and calcium concentration were associated with the differences in the DNA bacteriophage compositions between the sites (*p*-value = 0.003 and 0.008, respectively, α = 0.05). A redundancy analysis (afterwards called RDA; Figure 4) was carried out to analyse the variations of bacteriophage genera depending on the soil properties of the sites. The RDA was built over the two first dimensions, covering together 67.5% of the fitted variation. Among all the abiotic factors, soil pH was the explanatory variable that had a significant weight on the construction of the RDA (*p*-value = 0.008, α = 0.05). On the one hand, the axis RDA1 was significantly correlated at 88.4% with soil pH (*p*-value < 0.01, α = 0.05) and at 79.2% with calcium cation (*p*-value < 0.01, α = 0.05). The RDA2, on the other hand, was not significantly correlated with any of the tested soil properties.

Along the RDA1 axis, both soil pH and calcium concentration explained the sites distributions. Indeed, Weierbach and Retgenbusch were displayed apart from the other sites, as they were the most acidic soils (pH = 4.1 and 4.5, respectively) and had the lowest concentration in calcium (Ca^2+^ = 21 and 16 positive charges per kg of soil, respectively). The RDA showed a particular correlation between the sites of Weierbach and Retgenbusch and the genus *Borockvirus*. On the contrary, Pall 1, Huescherbach and Daerent presented the highest soil pH (pH = 6.8, 6.4 and 6.3, respectively) with relatively high concentrations of calcium (Ca^2+^ = 166, 126 and 87 positive charges per kg of soil, respectively). In terms of DNA bacteriophage genera, these soil properties (i.e., pH and Ca^2+^) showed a negative correlation with *Bingvirus* and *Scapunavirus*. Finally, Mollbach showed a particular distribution, related to the magnesium content, as indeed its soil presented the highest concentration of magnesium (Mg = 52 mg/100 g of soil). Additionally, *Saphexavirus* displayed a positive correlation specifically to the magnesium concentration.

### 3.4. Known Bacterial Populations and Connections with the Known DNA-Bacteriophage Communities

**Figure 5:**
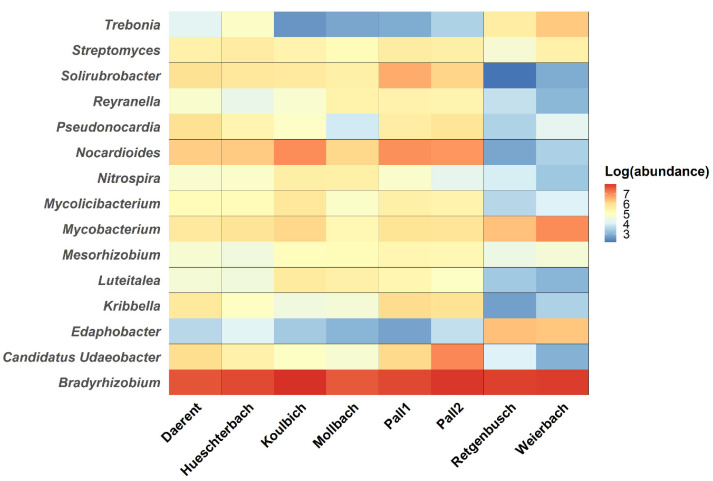
Heatmap of the most occurrent bacterial genera identified (selection with a log-abundance above 3) as a function of the sites. The gradient represents the log-transformed abundance of the genera, where red colour represents the most abundant genera and blue, the least ones.

As reported in Supplementary Table S1, bacterial sequences represented 74 to 77% of the total microbial sequences identified in the libraries. First, bacterial populations of all sites were mainly composed of the following phyla, *Acidobacteria*, *Actinobacteria*, *Proteobacteria* and *Verrucomicrobia* (see Supplementary Figure S1). As for the DNA bacteriophages, the taxonomical composition of the bacterial populations was then analysed at the genus level by selecting the most abundant bacteria (abundance above 1000 mapped reads per species, all sites included). Retgenbusch and Weierbach showed similar patterns in bacterial genera with the lowest soil bacterial diversity (H = 2.78 and 2.89, respectively) (see Supplementary Table S2) and similar bacterial composition as is visible in Figure 5.

**Figure 6:**
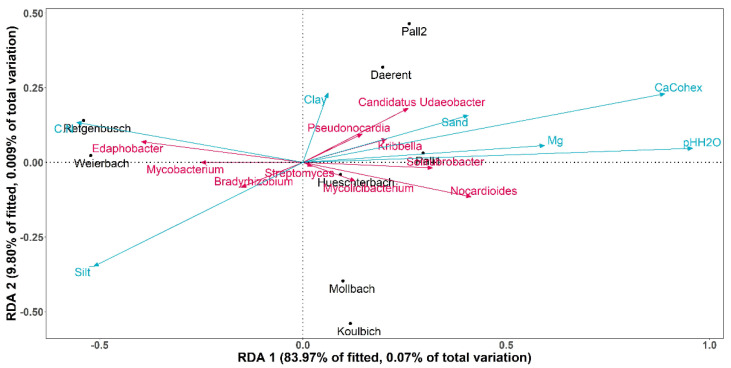
Biplot of redundancy analysis (RDA) on the 10 most abundant genera of bacteria in relation to the soil properties of each site. The pink vectors represent the bacterial genera, the blue vectors display the selected soil properties and the sites are shown in black. The RDA was built over the two first dimensions, with 83.97% of the fitted variations explained by RDA1 and 9.80% by RDA2. (C.N = carbon to nitrogen ratio; Mg = magnesium content; pHH_2_O = soil pH measured in water, CaCohex = calcium cation available).

In addition, these two sites showed differences in bacterial compositions from the other sites. Indeed, Regtenbusch and Weierbach showed higher abundance for the genera *Trebonia*, *Edaphobacter* and *Mycobacterium*, for which the relative abundances were lower in the other soils. Regarding the other genera, with the exception of *Streptomyces* and *Bradyrhizobium*, all of them showed high abundance for all sites except for Regtenbusch and Weierbach. Finally, *Streptomyces* and *Bradyrhizobium*, were detected in every soil with the highest abundance reported for the latter. The differences in bacterial community composition between the sites were studied through a PERMANOVA test, performed on the bacteria genera abundance. As noticed for the DNA bacteriophages, the vegetation (i.e., forest and grasslands) did not significantly impact the bacteria distribution (*p*-value = 0.121, α = 0.05). In addition, soil pH and calcium concentration also displayed significant effect on the bacterial compositions between the sites (*p*-value = 0.006 and 0.002, respectively, α = 0.05). Contrary to the observations on the DNA bacteriophages, magnesium concentration played a significant role on the bacterial composition (*p*-value = 0.027, α = 0.05). As for the DNA bacteriophage investigation, RDA was carried out to analyse the variations of bacterial genera depending on the same selected soil properties of the sites (Figure 6). The RDA was built over the two first dimensions, covering together 93.77% of the fitted variation. Among all the abiotic factors, soil pH was the sole explanatory variable that had a significant weight on the construction of the RDA (*p*-value = 0.01, α = 0.05). On one hand, the axis RDA1 was significantly correlated at 92.16% with soil pH (*p*-value < 0.01, α = 0.05) and at 79.21% with calcium (*p*-value < 0.01, α = 0.05). On the other hand, the RDA2 was not significantly correlated with any of the tested soil properties. Weierbach and Retgenbusch were found apart from all the other sites, and both related to only *Edaphobacter* and *Mycobacterium*.

**Figure 7:**
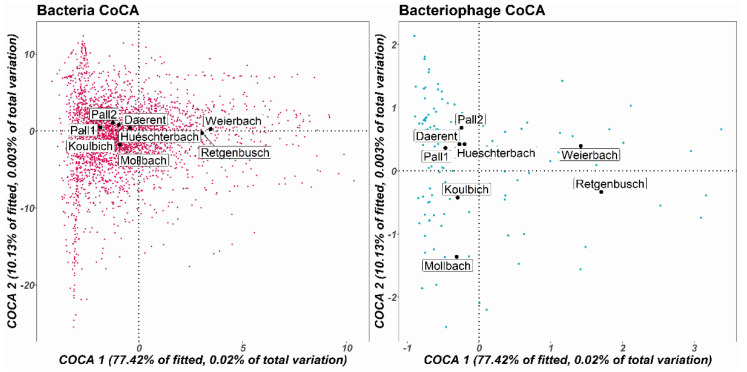
Symmetric co-correspondence analysis (Sym-CoCA) of both total bacteria and DNA bacteriophage community compositions. The bacterial genera are represented in pink and bacteriophage genera, in blue. Here, they were built over the two first dimensions, where 77.42% of the fitted covariation between the two matrices was explained by COCA1 and 10.13% by COCA2. The sites are displayed in black.

As DNA bacteriophages and bacteria are known for being exclusively linked to one another, the covariation of these two communities was analysed using a symmetric CoCA. This ordination method was built over the two first dimensions that covered 67.4% of the total covariation (Figure 7). It allows exploring the distribution of the whole known bacterial and DNA bacteriophage subset populations at the genera level by analysing both simultaneously. The patterns for both bacterial and DNA bacteriophage CoCA were similar and confirmed the distinct group formed by Weierbach and Retgenbusch. The latter was displayed offset from the other sites while Daerent, Pall 1, Pall 2, Mollbach and Hueschterbach were similar to each other on both bacterial and bacteriophage CoCA. A Mantel test using Pearson correlation carried on DNA bacteriophage and bacterial genera confirmed a strong positive correlation (R-value = 0.75, significance = 0.001).

## 4. Discussion

In our study, a viral concentration of approximately 10^9^ to 10^10^ VLPs per gram of dry soil was found, which is consistent with previous works [14,68,69]. Although VLP counts are not always considered to be a good representative of virus concentrations, the counts performed here were carried out in the same manner as previously on freshwater sediments using the FACSCalibur FCM table. This method was previously validated and provided similar VLP counts to those obtained by EFM [70]. A good correlation was found, highlighting the high sensitivity of the device and thus, allowing obtaining unambiguous signatures for VLPs by discriminating noise from VLPs (virus-like particles) to PLPs (prokaryote-like particles). It is noteworthy, however, that flow cytometry analysis does not allow identifying viruses or discriminating bacteriophages from the rest of the VLPS [71]. To draw a complete picture of the viral community, a taxonomic identification of the known DNA bacteriophage populations was complementarily performed through shotgun metagenomics and reference-based bioinformatics. The taxonomy was assigned using the nr database released in April 2022; however, it is noteworthy that the viral taxonomy built by the International Committee on Taxonomy of Viruses (ICTV) is nowadays updated according to the genetic similarities between bacteriophages, including reclassification of the families observed in the current study, such as *Siphoviridae*, *Myoviridae* and *Podoviridae*.

Our study showed *Caudovirales* as the most frequently identified order in all sites, while *Siphoviridae*, *Podoviridae* and *Myoviridae* were found as the most abundant families observed within the identified DNA bacteriophages, which is consistent with most of the literature that studied viral soil diversity [13,26,29]. However, the high proportion of these three families is rather the reflection of the taxonomy made available on the databases up to now, where most of the bacteriophage species are classified into these families [72]. For this reason, it is important to consider low taxonomic levels in diversity studies. In the current study, the viral genera obtained from the libraries did not display specific patterns related to the ecosystems (i.e., forests vs. grasslands). The three selected forest sites presented different vegetal covers (i.e., beech, spruce and oak) with diverse types of soil textures. The forest sites might not be sufficiently similar to constitute a common pattern in DNA bacteriophage diversity. However, several patterns in the known DNA bacteriophage distribution are distinguishable by considering the specific soil parameters. The soil pH as well as the calcium concentration significantly drove the ecological distribution of the known DNA bacteriophage communities among the different studied soils. More interestingly, the distribution of both DNA bacteriophage and bacterial communities in diverse types of soil were found to be strongly linked to one another and their distributions were explained by the same abiotic factors, i.e., soil pH and calcium concentration. Additionally, a complementary effect of magnesium concentration solely on the bacterial distribution was detected. The differences in microbial diversity were highlighted especially for the soils of Weierbach and Retgenbusch. Indeed, these two sites did not only display an acidic pH (around 4) with low calcium availability (<5 meq per 100 g of soil), but also low abundance and diversity of the known microbial populations at the genus level. While studying the known DNA bacteriophage populations, these same three soils were represented by the significant presence of one specific DNA bacteriophage genus. Indeed, *Saphexavirus*, *Borockvirus* and *Lessievirus* were highly abundant in the soil of Mollbach, Retgenbusch and Weierbach, respectively, compared to the other studied soils. Some studies reported a dominance of small ssDNA viruses rather than dsDNA-tailed bacteriophages in the soils [73,74,75]. In the present study, ssDNA bacteriophages (*Microviridae* and *Inoviridae*) were also detected, but in low frequency in comparison to dsDNA bacteriophages. Most ssDNA bacteriophages detected from environmental metagenomes belonged to the *Microviridae* family, for which all members infect a narrow range of hosts [76]. However, most ssDNA bacteriophages remain scarcely characterised within the taxonomy as *Inoviridae* represents 11% and *Microviridae* 2% of the ICTV database [77], reflecting a potential bias in the determination of their abundance in the soil samples.

Calcium is an important component participating in soil life. It plays a role in the soil structure, firstly as a binding agent in the aggregation of soil particles, secondly as a macronutrient in plant nutrition and thirdly as a factor facilitating soil permeability [78]. Then, calcium has a biological role in promoting an optimal environment to enhance soil microorganism activities [79]. Finally, calcium is highly related to soil pH. Indeed, soils with low pH generally display low calcium availability. As high concentrations of hydrogen ions displace calcium concentration from binding sites, it results in the leaching of “free” calcium, particularly in the more porous soils [80,81]. Such soils, such as the ones of Weierbach and Retgenbusch, i.e., low pH and calcium concentration, did display a low microbial diversity among the bacterial and DNA bacteriophage populations characterised in this study. On the contrary, Pall 1, Pall 2 and Daerent with much higher calcium concentrations (between 10 and 20 meq per 100 g of soil) and an ideal range of soil pH (between 6 and 7) showed higher microbial diversity. In laboratory experiments, divalent cations (such as calcium and magnesium) were found to interact with bacteriophage MS2 by increasing its attachment to the surface of river natural organic matter [82]. In soil, adsorption onto surface particles is the most important process implied in the bacteriophages-soil interactions [11]. This interaction is highly dependent upon the type of cations found in the environment. Calcium cation (positively charged) is highly involved in the adsorption of clay–humus complex (CHC), negatively charged on its surface, through electrostatic forces [83,84]. Bacteriophages can then adsorb onto the CHC by the presence of the cations on the surface. Indeed, bacteriophages are charged on their surface; however, this charge is highly dependent on pH. At neutral pH (7), most bacteriophages are negatively charged and can interact with the available cations [85]. Reducing the pH will induce some bacteriophage surfaces to turn into positive charges, according to their isoelectric point (pI). Once positive, bacteriophages can compete with cations to bind with the clay charges of the CHC. Indeed, bacteriophages tend to be more retained in soils rich in clay. The latter has a lower specific surface, due to large particles and a greater ion-exchange capacity, which highly adsorb bacteriophages [86]. Therefore, the combination between calcium cation and pH can play a role in the bacteriophage dynamic in soils. However, not only did these factors show a significant effect on the DNA bacteriophage distribution, but they also did on the bacterial distribution. Additionally, our results showed a strong positive correlation between both bacterial and DNA bacteriophages distribution among the sites, suggesting a strong interaction. Therefore, soil pH seems like the most significant factor in leading the known DNA bacteriophage distributions, directly or/and indirectly through the shaping of bacterial communities. Calcium cation availability might rather play a role in the dynamic of DNA bacteriophages, through their adsorption with the soil particles.

The advances in metagenomics and bioinformatics have enabled deeper investigations for the identification of viruses [87,88]; however, some technical issues remain in the analysis of soil viruses [89]. As viruses tend to adsorb on soil particles, virus elution and concentration steps are often required before performing metagenomics. Nevertheless, this step generally ends in low recovery efficiencies [90,91], as noticed in our results during the optimisation of the elution and concentration protocol. Indeed, even though the viral recovery was satisfactory to conduct a viral quantification using flow cytometry, it resulted in low DNA yield after nucleic acid extraction. Therefore, it affected the good quality of viral sequences obtained after conducting the shotgun metagenomics, leading us to carry out the DNA extraction directly from the soil samples. As the analysis is solely relying on virus-affiliated reads that can be obtained from total DNA metagenomes, few viral contigs were obtained with N50 comprising between 200 and 300 per library. This can limit the interpretation of relationships between viruses and other microbes within the same samples.

Much remains to be understood about the diversity, dynamics and roles of bacteriophages in soils and metagenomics approaches have only recently enabled increasing our knowledge, particularly under in situ conditions. In laboratory experiments, some physicochemical properties, especially pH and ion strength, influenced bacteriophage inactivation [92,93]. Our results seemed to show the effect of these similar factors on bacteriophage diversity in soils; however, it is noteworthy that the study was focused on the known fraction of the microbial populations in the diverse selected soils. The OTU-based approach is less dependent on reference databases but works by clustering sequences according to similarity thresholds (often set at a sequence similarity level above 97%) [94,95,96]. This approach does not identify the taxonomic species but is rather a proxy for them and has shown to be phylogenetically incoherent [94,95,96]. Therefore, further analyses using complementary molecular and bioinformatics methods, including untargeted approaches, could be applied to obtain a more complete picture of bacteriophage dynamics. Additionally, the investigation of the influence of certain abiotic factors on bacteriophage diversity based on what has already been characterised could open perspectives for the use of these species as, for instance, biotechnologies or in the development of new culture models for laboratory experiments.

## 5. Conclusions

In this study, the natural diversity and distribution of DNA bacteriophage populations in soils were explored as well as their relationship with the soil-dwelling bacterial populations. Through the complementary use of metagenomics, reference-based bioinformatics and multidimensional analyses, we identified the known fraction of DNA bacteriophage communities in forest and grassland soils and deciphered the influence of some soil properties and microbial communities on the DNA bacteriophage occurrence. Both soil pH and calcium content were observed to influence the distribution of DNA bacteriophage and bacterial populations. A strong positive correlation between DNA bacteriophage and bacterial communities was confirmed, suggesting in addition to soil pH and calcium content, bacteria are also involved. Therefore, our results add to the growing body of evidence that bacteriophages are likely to play key roles in soil ecology.

## Figures and Tables

**Table 1:** List of the samples with their GPS coordinates.

Sample Sites	Sample ID	Vegetation	GPS Coordinates
Weierbach	WPF12092019	Forest	(N) 49.831937 (E) 5.80341
Retgenbusch	RPF12092019	Forest	(N) 49.743436 (E) 5.834341
Daerent	DPF12092019	Forest	(N) 49.748226 (E) 5.856064
Koulbich	KPF12092019	Grassland	(N) 49.751942 (E) 5.826879
Hueschterbach	HPF12092019	Grassland	(N) 49.809914 (E) 5.863031
Pall 1	P1PF12092019	Grassland	(N) 49.74617 (E) 5.871577
Pall 2	P2PF12092019	Grassland	(N) 49.748935 (E) 5.881184
Mollbach	MPF12092019	Grassland	(N) 49.7347405 (E) 5.888961

**Table 2:** Physical-chemical properties of the eight studied soils: GWC: gravimetric water content, C_org_: organic carbon, N_tot_: total nitrogen, CEC: cation exchangeable capacity, S/T: cation saturation rate, Ca^2+^: calcium concentration.

Site Names	GWC	pH	C/N	C_org_	N_tot_	Mg	Na	P_2_O_5_	K_2_O	CEC	Ca^2+^	S/T	Textural Class	Clay	Silt	Sand
	%	-	-	%	%	mg per 100 g ds	mg per 100 g ds	mg per 100 g ds	mg per 100 g ds	Meq kg^−1^	Meq kg^−1^	%		%	%	%
Weierbach	24.6	4.1	18	9.7	0.54	3	2	5	13	85	21	42	CL	23.9	42.2	33.9
Retgenbusch	13.7	4.5	8.6	1.9	0.22	3	1	3	9	26	16	101	A	27.2	58.5	14.3
Daerent	15.8	6.3	10	3.9	0.39	46	2	4	21	171	126	107	SL	53.9	28.0	18.1
Koulbich	10.8	5.7	8.3	1.9	0.23	12	3	9	11	74	58	105	L	37.7	43.6	18.6
Hueschterbach	14.7	6.4	8.8	2.2	0.25	11	3	20	16	85	87	126	L	52.0	29.6	18.4
Pall 1	20.9	6.8	9.8	5.1	0.52	17	2	6	17	237	166	100	CL	24.2	42.6	33.2
Pall 2	16.3	6.2	8.8	3.6	0.41	31	3	7	15	172	151	115	CL	33.9	38.1	28.1
Mollbach	18.3	6.0	8.3	3.1	0.23	52	5	13	21	143	131	112	SL	28.1	52.5	19.4

ds = dry soil, meq kg: milliequivalent per kilogram (quantity of positive charges per kilogram); textural class: CL = clay loam; L = silt loam; SL = sandy loam; L = loam [67].

**Table 3:** Statistics of the metagenomic sequencing: raw data, assembly and mapping on the eight studied soils.

Sites	Number of Read Pairs	Yield in Mbp	Average Quality (Q-Score)	Number of Total Mapped Read Pairs	Percentage of Mapped Read Pairs	Number of Total Contigs	GC Content	N50
Daerent	45,506,199	12,929	35.6	36,248,481	79.66%	5,469,310	63.70%	271
Hueschterbach	42,222,600	13,046	35.88	36,987,700	87.60%	5,727,441	63.95%	269
Koulbich	64,930,676	18,587	35.53	49,069,306	75.57%	7,792,136	63.18%	268
Mollbach	50,983,302	14,522	35.63	35,064,164	68.77%	6,306,081	63.40%	264
Pall 1	46,905,743	13,397	35.62	40,072,879	85.43%	5,980,767	64.72%	273
Pall 2	56,510,613	16,136	35.6	48,535,078	85.89%	6,883,923	63.40%	272
Retgenbusch	49,316,386	13,878	35.59	46,692,284	94.68%	5,677,103	60.01%	295
Weierbach	57,936,000	16,650	35.22	57,518,714	99.28%	5,898,678	60.95%	323

Mbp = millions of base pairs; GC = guanine cytosine; N50 = the sequence length of the shortest contig at 50% of the total genome length; total references to total microbial metagenomes (i.e., eukaryote, archaea, bacteria and viruses).

**Table 4:** Shannon diversity index (H), evenness (E) and richness (S) on genus level of DNA bacteriophage from the known fraction of the population from the soil of the studied sites.

Sites	Shannon Index (H)	Evenness (E)	Species Richness (S)
Daerent	2.39	0.48	147
Hueschterbach	2.54	0.51	140
Koulbich	2.58	0.51	149
Mollbach	2.79	0.55	155
Pall 1	2.65	0.52	160
Pall 2	2.62	0.51	174
Retgenbusch	2.31	0.49	117
Weierbach	2.83	0.63	91

**Table 5:** PERMANOVA tests to analyse the influence of the selected abiotic factors on the distribution of the known DNA bacteriophage genera (α = 0.05).

Environmental Variables	*p*-Value
Vegetation	0.075
Ca^2+^	0.008 **
Carbon-to-nitrogen ratio	0.349
pH	0.003 **
Silt content	0.125
Sand content	0.764
Clay content	0.901
Mg content	0.083
GWC	0.846

Significance: ** *p* < 0.01.

## Data Availability

The raw sequence data generated and analysed within this study are available in the National Centre for Biotechnology Information Databases (Sequence Read Archive) under the BioProject number PRJNA771214.

## Supplementary Materials

The following supporting information can be downloaded at: https://www.mdpi.com/article/10.3390/microorganisms10071458/s1, Figure S1: Bar charts of the relative abundance (in %) of bacterial phylum (selected genera with an abundance above 100 occurrences) from the known fraction of all studied sites; Figure S2: Per base sequence quality from FastQC for read forward 1 (A) and read reverse 2 (B) of the sequencing ot the Weierbach soil; Table S1: Percentages of relative abundances identified for each taxonomic domain; Table S2: Shannon diversity index (H), evenness (E) and species richness (S) of bacteria populations in the soil of the studied sites; Table S3: Recovery of bacteriophage ΦX174 comparing three methods for mechanical viral detachment from the Weierbach soil; Table S4: Recovery of bacteriophage ΦX174 from the Weierbach soil comparing six different eluent solutions; Table S5: Recovery of bacteriophage ΦX174 from the Weierbach soil after concentration step, comparing Amicon with two different cut-off points (i.e., 30 kDa and 50 kDa); Table S6: Recovery of bacteriophage ΦX174 at each step of the elution and concentration protocol for each study site; Table S7: Summary of each step from the DNase treatment to the library preparation from viral concentrates and soil sample of the Weierbach site. References [97,98,99,100,101,102] are cited in the supplementary materials.

## References

[1] Lange M., Eisenhauer N., Sierra C.A., Bessler H., Engels C., Griffiths R.I., Mellado-Vázquez P.G., Malik A.A., Roy J., Scheu S. (2015). Plant Diversity Increases Soil Microbial Activity and Soil Carbon Storage. Nat. Commun..

[2] Vezzani F.M., Anderson C., Meenken E., Gillespie R., Peterson M., Beare M.H. (2018). The Importance of Plants to Development and Maintenance of Soil Structure, Microbial Communities and Ecosystem Functions. Soil Tillage Res..

[3] Eldridge D., James A. (2009). Soil-disturbance by Native Animals Plays a Critical Role in Maintaining Healthy Australian Landscapes. Ecol. Manag. Restor..

[4] Lou X., Zhao J., Lou X., Xia X., Feng Y., Li H. (2021). The Biodegradation of Soil Organic Matter in Soil-Dwelling Humivorous Fauna. Front. Bioeng. Biotechnol..

[5] Odelade K.A., Babalola O.O. (2019). Bacteria, Fungi and Archaea Domains in Rhizospheric Soil and Their Effects in Enhancing Agricultural Productivity. Int. J. Environ. Res. Public Health.

[6] Rashid M.I., Mujawar L.H., Shahzad T., Almeelbi T., Ismail I.M.I., Oves M. (2016). Bacteria and Fungi Can Contribute to Nutrients Bioavailability and Aggregate Formation in Degraded Soils. Microbiol. Res..

[7] Buerkert A., Joergensen R.G., Ludwig B., Schlecht E. (2012). Chapter 18—Nutrient and Carbon Fluxes in Terrestrial Agro-Ecosystems. Marschner’s Mineral Nutrition of Higher Plants.

[8] Ortiz A., Sansinenea E. (2022). The Role of Beneficial Microorganisms in Soil Quality and Plant Health. Sustainability.

[9] Beule L., Vaupel A., Moran-Rodas V.E. (2022). Abundance, Diversity, and Function of Soil Microorganisms in Temperate Alley-Cropping Agroforestry Systems: A Review. Microorganisms.

[10] Armon R., Witzany G. (2011). Soil Bacteria and Bacteriophages. Biocommunication in Soil Microorganisms.

[11] Kimura M., Jia Z.J., Nakayama N., Asakawa S. (2008). Ecology of Viruses in Soils: Past, Present and Future Perspectives. Soil Sci. Plant Nutr..

[12] Coyne M.S. (1996). A Cartoon History of Soil Microbiology. J. Nat. Resour. Life Sci. Educ..

[13] Williamson K.E., Fuhrmann J.J., Wommack K.E., Radosevich M. (2017). Viruses in Soil Ecosystems: An Unknown Quantity Within an Unexplored Territory. Annu. Rev. Virol..

[14] Williamson K.E., Corzo K.A., Drissi C.L., Buckingham J.M., Thompson C.P., Helton R.R. (2013). Estimates of Viral Abundance in Soils Are Strongly Influenced by Extraction and Enumeration Methods. Biol. Fertil. Soils.

[15] Bertrand I., Schijven J.F., Sánchez G., Wyn-Jones P., Ottoson J., Morin T., Muscillo M., Verani M., Nasser A., de Roda Husman A.M. (2012). The Impact of Temperature on the Inactivation of Enteric Viruses in Food and Water: A Review. J. Appl. Microbiol..

[16] Corapcioglu M.Y., Vogel J.R., Munster C.L., Pillai S.D., Dowd S., Wang S. (2006). Virus Transport Experiments in a Sandy Aquifer. Water Air Soil Pollut..

[17] Fauvel B., Cauchie H.M., Gantzer C., Ogorzaly L. (2019). Influence of Physico-Chemical Characteristics of Sediment on the in Situ Spatial Distribution of F-Specific RNA Phages in the Riverbed. FEMS Microbiol. Ecol..

[18] Jurczak-Kurek A., Gasior T., Nejman-Faleńczyk B., Bloch S., Dydecka A., Topka G., Necel A., Jakubowska-Deredas M., Narajczyk M., Richert M. (2016). Biodiversity of Bacteriophages: Morphological and Biological Properties of a Large Group of Phages Isolated from Urban Sewage. Sci. Rep..

[19] Feng Y.Y., Ong S.L., Hu J.Y., Tan X.L., Ng W.J. (2003). Effects of PH and Temperature on the Survival of Coliphages MS2 and Qβ. J. Ind. Microbiol. Biotechnol..

[20] Nishide M., Tsujimoto K., Uozaki M., Ikeda K., Yamasaki H., Koyama A.H., Arakawa T. (2011). Effects of Electrolytes on Virus Inactivation by Acidic Solutions. Int. J. Mol. Med..

[21] Taj M.K., Ling J.X., Bing L.L., Qi Z., Taj I., Hassani T.M., Samreen Z., Yunlin W. (2014). Effect of Dilution, Temperature and PH on the Lysis Activity of T4 Phage against E. Coli BL21. J. Anim. plant Sci..

[22] Cuadros J. (2017). Clay Minerals Interaction with Microorganisms: A Review. Clay Miner..

[23] Park J.A., Kang J.K., Kim J.H., Kim S.B., Yu S., Kim T.H. (2015). Bacteriophage Removal in Various Clay Minerals and Clay-Amended Soils. Environ. Eng. Res..

[24] Harvey R.W., Ryan J.N. (2004). Use of PRD1 Bacteriophage in Groundwater Viral Transport, Inactivation, and Attachment Studies. FEMS Microbiol. Ecol..

[25] Williamson K.E., Radosevich M., Wommack K.E. (2005). Abundance and Diversity of Viruses in Six Delaware Soils. Appl. Environ. Microbiol..

[26] Leroy M., Prigent M., Dutertre M., Confalonieri F., Dubow M. (2008). Bacteriophage Morphotype and Genome Diversity in Seine River Sediment. Freshw. Biol..

[27] Parmar K., Dafale N., Pal R., Tikariha H., Purohit H. (2018). An Insight into Phage Diversity at Environmental Habitats Using Comparative Metagenomics Approach. Curr. Microbiol..

[28] Mohiuddin M., Schellhorn H.E. (2015). Spatial and Temporal Dynamics of Virus Occurrence in Two Freshwater Lakes Captured through Metagenomic Analysis. Front. Microbiol..

[29] Gong Z., Liang Y., Wang M., Jiang Y., Yang Q., Xia J., Zhou X., You S., Gao C., Wang J. (2018). Viral Diversity and Its Relationship With Environmental Factors at the Surface and Deep Sea of Prydz Bay, Antarctica. Front. Microbiol..

[30] Narr A., Nawaz A., Wick L.Y., Harms H., Chatzinotas A. (2017). Soil Viral Communities Vary Temporally and along a Land Use Transect as Revealed by Virus-like Particle Counting and a Modified Community Fingerprinting Approach (FRAPD). Front. Microbiol..

[31] Trubl G., Jang H.B., Roux S., Emerson J.B., Solonenko N., Vik D.R., Solden L., Ellenbogen J., Runyon A.T., Bolduc B. (2018). Soil Viruses Are Underexplored Players in Ecosystem Carbon Processing. mSystems.

[32] Cao M.-M., Liu S.-Y., Bi L., Chen S.-J., Wu H.-Y., Ge Y., Han B., Zhang L.-M., He J.-Z., Han L.-L. (2022). Distribution Characteristics of Soil Viruses Under Different Precipitation Gradients on the Qinghai-Tibet Plateau. Front. Microbiol..

[33] Han L.L., Yu D.T., Bi L., Du S., Silveira C., Cobián Güemes A.G., Zhang L.M., He J.Z., Rohwer F. (2022). Distribution of Soil Viruses across China and Their Potential Role in Phosphorous Metabolism. Environ. Microbiome.

[34] Koskella B., Brockhurst M.A. (2014). Bacteria–Phage Coevolution as a Driver of Ecological and Evolutionary Processes in Microbial Communities. FEMS Microbiol. Rev..

[35] Fazzino L., Anisman J., Chacón J.M., Heineman R.H., Harcombe W.R. (2020). Lytic Bacteriophage Have Diverse Indirect Effects in a Synthetic Cross-Feeding Community. ISME J..

[36] Sullivan M.B., Weitz J.S., Wilhelm S. (2017). Viral Ecology Comes of Age. Environ. Microbiol. Rep..

[37] Klimenko A.I., Matushkin Y.G., Kolchanov N.A., Lashin S.A. (2016). Bacteriophages Affect Evolution of Bacterial Communities in Spatially Distributed Habitats: A Simulation Study. BMC Microbiol..

[38] Ashelford K.E., Day M.J., Fry J.C. (2003). Elevated Abundance of Bacteriophage Infecting Bacteria in Soil. Appl. Environ. Microbiol..

[39] Braga L.P.P., Spor A., Kot W., Breuil M.C., Hansen L.H., Setubal J.C., Philippot L. (2020). Impact of Phages on Soil Bacterial Communities and Nitrogen Availability under Different Assembly Scenarios. Microbiome.

[40] Marx S., Flammang F. (2015). La Cartographie Des Sols Au Grand-Duché de Luxembourg Version Provisoire_V4.

[41] Heffernan A.L., Aylward L.L., Toms L.M.L., Sly P.D., Macleod M., Mueller J.F. (2014). Pooled Biological Specimens for Human Biomonitoring of Environmental Chemicals: Opportunities and Limitations. J. Expo. Sci. Environ. Epidemiol..

[42] Caudill S.P. (2010). Characterizing Populations of Individuals Using Pooled Samples. J. Expo. Sci. Environ. Epidemiol..

[43] Roy K., Ghosh D., DeBruyn J.M., Dasgupta T., Wommack K.E., Liang X., Wagner R.E., Radosevich M. (2020). Temporal Dynamics of Soil Virus and Bacterial Populations in Agricultural and Early Plant Successional Soils. Front. Microbiol..

[44] AFNOR (2021). ISO 10390: Sols, Biodéchets Traités et Boues—Détermination Du PH.

[45] VDLUFA (2016). Methodenbuch A5.1.1: Bestimmung des PH-Wertes.

[46] VDLUFA (2012). Methodenbuch A6.2.1.1: Bestimmung von Phosphor und Kalium im Calcium-Acetat-Lactat-Auszug.

[47] VDLUFA (1997). Methodenbuch A6.2.1.7: Bestimmung von Pflanzenverfügbarem Kalium und Natrium im Calciumchloridauszug auf Gewichtsbasis.

[48] AFNOR (1995). ISO 10694: Qualité du Sol–Dosage du Carbone Organique et du Carbon Total Après Combustion Sèche (Analyse Élémentaire).

[49] AFNOR (1998). ISO 13878: Qualité du Sol–Détermination de la Teneur Totale en Azote par Combustion Sèche (“Analyse Élémentaire”).

[50] AFNOR (2007). ISO 23470: Qualité Du Sol–Détermination de La Capacité D’échange Cationique (CEC) Effective et des Cations Échangeables à L’aide d’une Solution de Trichlorure de Cobaltihexammine.

[51] Marx S., Flammang F. (2018). Cartographie des Sols au Grand-Duché de Luxembourg.

[52] Jacquet S., Dorigo U., Personnic S. (2013). A Few Tests Prior to Flow Cytometry and Epifluorescence Analyses of Freshwater Bacterioand Virioplankton Communities. Flow Cytom. Princ. Methodol. Appl..

[53] Bolger A.M., Lohse M., Usadel B. (2014). Trimmomatic: A Flexible Trimmer for Illumina Sequence Data. Bioinformatics.

[54] Nurk S., Meleshko D., Korobeynikov A., Pevzner P.A. (2017). MetaSPAdes: A New Versatile Metagenomic Assembler. Genome Res..

[55] Steinegger M., Söding J. (2017). MMseqs2 Enables Sensitive Protein Sequence Searching for the Analysis of Massive Data Sets. Nat. Biotechnol..

[56] Mirdita M., Steinegger M., Breitwieser F., Sö Ding J., Levy Karin E. (2021). Fast and Sensitive Taxonomic Assignment to Metagenomic Contigs. Bioinformatics.

[57] Li H. (2018). Minimap2: Pairwise Alignment for Nucleotide Sequences. Bioinformatics.

[58] Danecek P., Bonfield J.K., Liddle J., Marshall J., Ohan V., Pollard M.O., Whitwham A., Keane T., McCarthy S.A., Davies R.M. (2021). Twelve Years of SAMtools and BCFtools. Gigascience.

[59] Palermo C.N., Fulthorpe R.R., Saati R., Short S.M. (2019). Metagenomic Analysis of Virus Diversity and Relative Abundance in a Eutrophic Freshwater Harbour. Viruses.

[60] Zhao Y., Li M.C., Konaté M.M., Chen L., Das B., Karlovich C., Williams P.M., Evrard Y.A., Doroshow J.H., McShane L.M. (2021). TPM, FPKM, or Normalized Counts? A Comparative Study of Quantification Measures for the Analysis of RNA-Seq Data from the NCI Patient-Derived Models Repository. J. Transl. Med..

[61] RStudio Team (2020). RStudio: Integrated Development for R. RStudio.

[62] Thukral A.K. (2017). A Review on Measurement of Alpha Diversity in Biology. Agric. Res. J..

[63] Oksanen J., Blanchet F.G., Friendly M., Kindt R., Legendre P., McGlinn D., Minchin P.R., O’Hara R.B., Simpson G.L., Solymos P. (2020). vegan: Community Ecology Package. R Package Version 2.5-7. https://CRAN.R-project.org/package=vegan.

[64] Mulder C.P.H., Bazeley-White E., Dimitrakopoulos P.G., Hector A., Scherer-Lorenzen M., Schmid B. (2004). Species Evenness and Productivity in Experimental Plant Communities. Oikos.

[65] Moore J.C. (2013). Diversity, Taxonomic versus Functional. Encyclopedia of Biodiversity.

[66] Socolar J.B., Gilroy J.J., Kunin W.E., Edwards D.P. (2016). How Should Beta-Diversity Inform Biodiversity Conservation?. Trends Ecol. Evol..

[67] USDA (United States Department of Agriculture, Natural resources conservation service, Soil Texture Calculator. https://www.nrcs.usda.gov/wps/portal/nrcs/detail/soils/survey/?cid=nrcs142p2_054167.

[68] Pratama A.A., van Elsas J.D. (2018). The ‘Neglected’ Soil Virome—Potential Role and Impact. Trends Microbiol..

[69] Williamson K.E., Wommack K.E., Radosevich M. (2003). Sampling Natural Viral Communities from Soil for Culture-Independent Analyses. Appl. Environ. Microbiol..

[70] Duhamel S., Jacquet S. (2006). Flow Cytometric Analysis of Bacteria- and Virus-like Particles in Lake Sediments. J. Microbiol. Methods.

[71] Colombet J., Billard H., Viguès B., Balor S., Boulé C., Geay L., Benzerara K., Menguy N., Ilango G., Fuster M. (2019). Discovery of High Abundances of Aster-Like Nanoparticles in Pelagic Environments: Characterization and Dynamics. Front. Microbiol..

[72] Turner D., Kropinski A.M., Adriaenssens E.M. (2021). A Roadmap for Genome-Based Phage Taxonomy. Viruses.

[73] Kim K.H., Chang H.W., Nam Y.D., Roh S.W., Kim M.S., Sung Y., Jeon C.O., Oh H.M., Bae J.W. (2008). Amplification of Uncultured Single-Stranded DNA Viruses from Rice Paddy Soil. Appl. Environ. Microbiol..

[74] Reavy B., Swanson M.M., Cock P.J.A., Dawson L., Freitag T.E., Singh B.K., Torrance L., Mushegian A.R., Taliansky M. (2015). Distinct Circular Single-Stranded DNA Viruses Exist in Different Soil Types. Appl. Environ. Microbiol..

[75] Grujcic V., Nuy J.K., Salcher M.M., Shabarova T., Kasalicky V., Boenigk J., Jensen M., Simek K. (2018). Cryptophyta as Major Bacterivores in Freshwater Summer Plankton. ISME J..

[76] Cherwa J.E., Fane B.A. (2011). Microviridae: Microviruses and Gokushoviruses. eLS.

[77] Székely A.J., Breitbart M. (2016). Single-Stranded DNA Phages: From Early Molecular Biology Tools to Recent Revolutions in Environmental Microbiology. FEMS Microbiol. Lett..

[78] Wuddivira M.N., Camps-Roach G. (2007). Effects of Organic Matter and Calcium on Soil Structural Stability. Eur. J. Soil Sci..

[79] Zavarzin G.A. (2002). Microbial Geochemical Calcium Cycle. Microbiology.

[80] Whittinghill K.A., Hobbie S.E. (2011). Effects of PH and Calcium on Soil Organic Matter Dynamics in Alaskan Tundra. Biogeochemistry.

[81] Ng J.F., Ahmed O.H., Jalloh M.B., Omar L., Kwan Y.M., Musah A.A., Poong K.H. (2022). Soil Nutrient Retention and PH Buffering Capacity Are Enhanced by Calciprill and Sodium Silicate. Agronomy.

[82] Pham M., Mintz E.A., Nguyen T.H. (2009). Deposition Kinetics of Bacteriophage MS2 to Natural Organic Matter: Role of Divalent Cations. J. Colloid Interface Sci..

[83] Brydon J.E., Sowden F.J. (2011). A Study o the Clay-Humus Complexes of a Chernozemic and a Podzol Soil. J. Soil Sci..

[84] Xu X., Li Y., Hu X., Xie G., Xu H., Gao M., Zhang X., Zhang R., Tang C., Hu X. (2022). Effect of Humic Acid on the Adsorption/Desorption Behaviors of Trivalent Chromium on Calcium Modified Montmorillonite and Kaolinite. ChemistrySelect.

[85] Sadeghi G., Behrends T., Schijven J.F., Hassanizadeh S.M. (2013). Effect of Dissolved Calcium on the Removal of Bacteriophage PRD1 during Soil Passage: The Role of Double-Layer Interactions. J. Contam. Hydrol..

[86] McLeod M., Aislabie J., Smith J., Fraser R., Roberts A., Taylor M. (2001). Viral and Chemical Tracer Movement through Contrasting Soils. J. Environ. Qual..

[87] Fernandez-Cassi X., Rusinol M., Martinez-Puchol S. (2018). Viral Concentration and Amplification from Human Serum Samples Prior to Application of Next-Generation Sequencing Analysis. The Human Virome: Methods and Protocols.

[88] Zhang Y.Z., Shi M., Holmes E.C. (2018). Using Metagenomics to Characterize an Expanding Virosphere. Cell.

[89] Trubl G., Hyman P., Roux S., Abedon S.T. (2020). Coming-of-Age Characterization of Soil Viruses: A User’s Guide to Virus Isolation, Detection within Metagenomes, and Viromics. Soil Syst..

[90] Baer A., Kehn-Hall K. (2014). Viral Concentration Determination Through Plaque Assays: Using Traditional and Novel Overlay Systems. J. Vis. Exp..

[91] Van Twest R., Kropinski A.M., Clokie M.R.J., Kropinski A.M. (2009). Bacteriophages Enrichment from Water and Soil.

[92] Walshe G.E., Pang L., Flury M., Close M.E., Flintoft M. (2010). Effects of PH, Ionic Strength, Dissolved Organic Matter, and Flow Rate on the Co-Transport of MS2 Bacteriophages with Kaolinite in Gravel Aquifer Media. Water Res..

[93] Furiga A., Pierre G., Glories M., Aimar P., Roques C., Causserand C., Berge M. (2011). Effects of Ionic Strength on Bacteriophage MS2 Behavior and Their Implications for the Assessment of Virus Retention by Ultrafiltration Membranes. Appl. Environ. Microbiol..

[94] Fierer N., Breitbart M., Nulton J., Salamon P., Lozupone C., Jones R., Robeson M., Edwards R.A., Felts B., Rayhawk S. (2007). Metagenomic and Small-Subunit RRNA Analyses Reveal the Genetic Diversity of Bacteria, Archaea, Fungi, and Viruses in Soil. Appl. Environ. Microbiol..

[95] Nguyen N.P., Warnow T., Pop M., White B. (2016). A Perspective on 16S RRNA Operational Taxonomic Unit Clustering Using Sequence Similarity. NPJ Biofilms Microbiomes.

[96] Schloss P.D., Westcott S.L. (2011). Assessing and Improving Methods Used in Operational Taxonomic Unit-Based Approaches for 16S RRNA Gene Sequence Analysis. Appl. Environ. Microbiol..

[97] Chen L., Xun W., Sun L., Zhang N., Shen Q., Zhang R. (2014). Effect of Different Long-Term Fertilization Regimes on the Viral Community in an Agricultural Soil of Southern China. Eur. J. Soil Biol..

[98] Doan T.T., Bouvier C., Bettarel Y., Bouvier T., Henry-des-Tureaux T., Janeau J.L., Lamballe P., Van Nguyen B., Jouquet P. (2014). Influence of Buffalo Manure, Compost, Vermicompost and Biochar Amendments on Bacterial and Viral Communities in Soil and Adjacent Aquatic Systems. Appl. Soil Ecol..

[99] Swanson M.M., Fraser G., Daniell T.J., Torrance L., Gregory P.J., Taliansky M. (2009). Viruses in Soils: Morphological Diversity and Abundance in the Rhizosphere. Ann. Appl. Biol..

[100] Ateba C.N., Akindolire M.A. (2019). Isolation and Characterisation of Bacteriophages with Lytic Activity Against Virulent Escherichia Coli O157:H7: Potential Bio-Control Agents. Preprints.

[101] Gupta V., Saxena H.M. (2017). Isolation and Characterization of BpL1, a Broad Acting Lytic Bacteriophage against Brucella. Int. J. Curr. Microbiol. Appl. Sci..

[102] Hyman P. (2019). Phages for Phage Therapy: Isolation, Characterization, and Host Range Breadth. Pharmaceuticals.

