# Supplementary material for: Soil pH, Calcium Content and Bacteria as Major Factors Responsible for the Distribution of the Known Fraction of the DNA Bacteriophage Populations in Soils of Luxembourg
Source: Microorganisms. 2022 Jul 19;10(7):1458. doi: 10.3390/microorganisms10071458 (PMC9321959; doi:10.3390/microorganisms10071458)
Supplement: Supplementary file 1 [file microorganisms-10-01458-s001.zip › Supplementary_FigureS1.pdf]

microorganisms

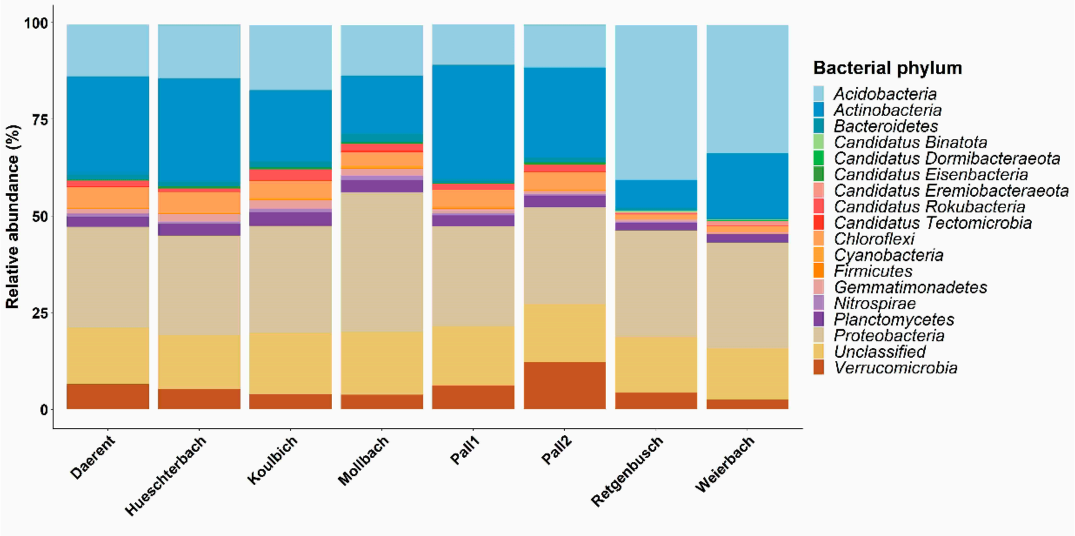

Supplementary Figure S1: Bar charts of the relative abundance (in %) of bacterial phylum (selected genera with an abundance above 100 occurrences) from the known fraction of all studied sites.
