# Supplementary material for: Soil pH, Calcium Content and Bacteria as Major Factors Responsible for the Distribution of the Known Fraction of the DNA Bacteriophage Populations in Soils of Luxembourg
Source: Microorganisms. 2022 Jul 19;10(7):1458. doi: 10.3390/microorganisms10071458 (PMC9321959; doi:10.3390/microorganisms10071458)
Supplement: Supplementary file 1 [file microorganisms-10-01458-s001.zip › Supplementary_material_v1.pdf]

Supplementary protocol

Section S1: Viral elution and concentration protocol

Materials and method

To optimise and validate our isolation method of viruses from other possible soil fractions (e.g. bacteria, eukaryotes, dust, soil particles…), an elution and concentration protocol was optimised on the soil of the Weierbach site, using bacteriophage ΦX174 (*Microviridae*) as surrogate [97–104]. Feed solutions of ΦX174 were produced with quantities comprised between 1.45 and 2.35 x 10^9 PFU (Plaque-Forming Unit) and spiked into the tested soil samples. The experiment was conducted in triplicate for each soil. The optimisation included the experimentation of three different parts of the protocol: mechanical detachment, chemical detachment, cell debris cleaning and concentration process. Regarding the detachment of the viral particles from the soil matrices, a combination of mechanical and chemical actions was assessed to increase the viral recovery rate. The mechanical detachment was explored through three options: (i) sonication during 3 minutes at 4°C (Sonicator S300 Elmasonic, 37 kHz, 300 W), (ii) bead-beating for 3 minutes at room temperature, 15 Hz (100-240V/60Hz, Retsch, Germany), and (iii) the absence of any mechanical process. In parallel, a chemical detachment was carried out investigating six eluent solutions, selected according to the literature [97,99,101]: (i) beef extract 3% mixed with glycine 0.05M (BG) at pH = 9.5, (ii) beef extract 10% (B10%) at pH = 9, (iii) glycine 0.25M (Gly) at pH = 8, (iv) potassium citrate (PC) at pH = 7, (v) sodium pyrophosphate (SP) at pH = 7, (vi) phosphate-buffered saline (PBS) at pH = 7. Regarding the viral concentration, an ultracentrifugal filtration was performed using an Amicon Ultra-15 (UFC905024, Merck Millipore Ltd.) with two tested cut-off points (30 kD and 50 kD). The feed solutions of ΦX174, as well as the recovered solutions, at every step were enumerated using the double agar layer assay according to the standard procedures ISO 10705-2:2001, and the Log Reduction Value (LRV) was estimated for each experimental condition as follow:

$$LRV = \log\left(\frac{Qr}{Qf}\right)$$

where Q_f and Q_r are the quantities of ΦX174 in the feed and recovered solutions, respectively. The protocol was then applied to the seven remaining sites for which the LRV was calculated as described above in the validation process of the optimisation.

Results

Viral mechanical detachment

The results regarding the optimisation of the viral mechanical detachment step are reported in Table S3. The sonication as well as the bead-beater methods revealed a loss in ΦX174 quantity of 0.7 log while the absence of these two methods in the protocol induced a loss of 0.8 log. According to the non-parametric test Kruskal-Wallis One Way ANOVA on rank, no significant difference was detected between all three methods ($p = 0.07$, $\alpha = 0.05$). As the two mechanical methods did not significantly allow improving the recovery of ΦX174 particles during the viral detachment, none of them was kept. In addition, the sonication method is known to be non-homogeneous in the spread of the sonic waves.

Table S3: Recovery of bacteriophage ΦX174 comparing three methods for mechanical viral detachment from the Weierbach soil. A Kruskal-Wallis one-way ANOVA on ranks, followed by a Dunn test, was performed on the results of the three methods to analyse their effectiveness on the elution of ΦX174 compared to the spiking solution.

Solutions	ΦX174 quantity Mean ± sd (PFU)	LRV Mean ± sd	p-value
Spiking	$2.05 \times 10^9 \pm 5.5 \times 10^8$	-	-
Sonication	$4.41 \times 10^8 \pm 1.62 \times 10^8$	- 0.70 ± 0.19	NS
Bead-beater	$4.16 \times 10^8 \pm 5.59 \times 10^7$	- 0.70 ± 0.06	NS
Nothing	$3.26 \times 10^8 \pm 4.89 \times 10^7$	- 0.80 ± 0.07	NS

LRV = Log Reduction Value; NS = Not significant

Viral chemical detachment

The second optimised method was viral chemical detachment for which the results are reported in Table S4. Overall, a reduction in ΦX174 quantity of more than 1 log from the spiking was observed for glycine, PBS, PC and SP solutions. In contrary, BG and B10%, showed a reduction below 1 log, with a better recovery for the mix beef extract and glycine than for the beef extract alone (LRV = -0.47 log vs. LRV = -0.72 log, respectively). A non-parametric test (Kruskal-Wallis one way ANOVA on rank) confirmed significant differences ($p = 0.01082$, $\alpha = 0.05$) between the different eluent solutions. More precisely, a significant difference was noticed for PBS, PC, SP, and Gly compared to the feed solution (p-value = 0.0057, 0.0211, 0.0350 and 0.0005, respectively). In contrary, B10% and BG did not show significant reduction in ΦX174 quantity compared to the feed solution (p-value = 0.2357 and 0.5532, respectively). In addition to the 1.5-fold decrease in the viral recovery of BG compared to B10%, carrying out the molecular technique after using BG is preferred from B10% since the amount of beef extract in much lower in the BG (3%) than in the B10% (10%). Indeed, the beef extract present in the eluent solution can bring DNA contamination during the metagenomics analyses. The eluent solution beef extract (3%) and glycine (0.05M) was thus selected.

Table S4: Recovery of bacteriophage ΦX174 from the Weierbach soil comparing six different eluent solutions. B10% = Beef extract 10% (pH = 9); BG = Beef extract 3% + Glycine 0.05M (pH = 9.5); Gly = Glycine 0.25M (pH = 8); PBS = Phosphate-buffered saline (pH = 7); PC = Potassium citrate (pH = 7); SP = Sodium pyrophosphate (pH = 7). A Kruskal-Wallis one-way ANOVA on ranks, followed by a Dunn test, was performed on the results of each eluent solution to analyse their impact on the recovery of ΦX174 compared to the spiking solution.

Solutions	ΦX174 quantity Mean ± sd (PFU	LRV Mean ± sd	p-value
Spiking	$2.35 \times 10^9 \pm 2.12 \times 10^8$	-	-
B10%	$4.51 \times 10^8 \pm 1.03 \times 10^8$	- 0.72 ± 0.11	0.2357
BG	$7.97 \times 10^8 \pm 1.00 \times 10^8$	- 0.47 ± 0.06	0.5532
GLY	$5.34 \times 10^7 \pm 2.32 \times 10^7$	- 1.67 ± 0.19	0.0005***
PBS	$9.43 \times 10^7 \pm 2.73 \times 10^7$	- 1.41 ± 0.12	0.0057**
PC	$1.16 \times 10^8 \pm 1.43 \times 10^7$	- 1.31 ± 0.05	0.0211*
SP	$1.23 \times 10^8 \pm 1.80 \times 10^7$	- 1.28 ± 0.06	0.0350*

Significance: ***p<0.001; ** p<0.01; * p<0.05. sd = standard deviation; LRV = Log Reduction Value; PFU = Plaque forming unit.

Concentration of viral particles

The average quantities of ΦX174 recovered after concentration were quantified for both concentrates and eluate (Amicons 50 kDa & 30 kDa) and are reported in Table S5.

After centrifugation, a reduction of 0.47 log was observed. After concentration by ultrafiltration, the log reductions of both Amicons 50 kDa & 30 kDa were similar with -0.89 log and -0.81 log, respectively and no significant difference with the feed solutions (p-value = 1.000 and 0.955, respectively). In contrast, the eluates showed different patterns between the two tested Amicons. The quantification of ΦX174 in the eluate allowed defining the number of lost bacteriophages. The eluate of the Amicon 50kDa was revealed to have a significant difference (p = 0.006) with a reduction of 8.3 log from the feed solution. However, no significant loss was observed in the eluate of the Amicon 30 kDa (LRV = -4.79 log, p-value = 0.055). Indeed, the quantity of bacteriophages in the eluate of the Amicon 30 kDa was found 4-log higher than in the Amicon 50 kDa, meaning that a fraction of ΦX174 was not properly recovered in the concentrate of the former.

Table S5: Recovery of bacteriophage ΦX174 from the Weierbach soil after concentration step, comparing Amicon with two different cut-off points (i.e., 30 kDa and 50 kDa). A Kruskal-Wallis one-way ANOVA on ranks, followed by a Dunn test, was performed on the results of each eluent solution to analyse their impact on the recovery of ΦX174 compared to the feed solution.

Solutions	ΦX174 quantity Mean ± sd (PFU)	LRV Mean ± sd	p-value
Spiking	$2.35 \times 10^9 \pm 2.12 \times 10^8$	-	-
Centrifuge	$7.97 \times 10^8 \pm 8.18 \times 10^7$	- 0.47 ± 0.04	1.000
Concentrate 50 kDa	$3.56 \times 10^8 \pm 1.66 \times 10^8$	- 0.89 ± 0.27	1.000
Eluate 50 kDa	$9.00 \times 10^0 \pm 8.29 \times 10^0$	- 8.3 ± 0.23	0.006**
Concentrate 30 kDa	$4.50 \times 10^8 \pm 3.29 \times 10^8$	- 0.81 ± 0.27	0.955
Eluate 30 kDa	$8.39 \times 10^4 \pm 1.82 \times 10^4$	- 4.79 ± 0.51	0.055

Significance: ** p<0.01; sd = standard deviation; LRV = Log Reduction Value; PFU = Plaque forming unit.

It is noteworthy that a clean-up treatment with chloroform (1:20 v/v) was performed to remove any remaining cellular debris from the final viral concentrates. After vortexing for 5 min, the solutions were centrifuged at 10,000 g for 5 min. As a result, contamination was observed during the plaque assays and therefore did not allow successful viral recovery. In general, bacteriophages show good stability in chloroform, provided that the sensitivity of the bacteriophage is known [102]. Indeed, in some cases it has been found that chloroform can potentially cause inactivation of some bacteriophages [103,104]. For these reasons, this clean-up treatment was not retained for the final protocol.

Comparison for all sites: protocol validation

Once the optimisation for the viral elution and concentration was successfully carried out on the soil of the Weierbach, the protocol was performed on the samples of the remaining soils (i.e., Daerent, Retgenbusch, Pall 1, Pall 2, Hueschterbach, Koulbich and Mollbach). The results obtained were then reported on the Table S6, as the average quantity of **Φ**X174 recovered after each step (i.e., chemical elution and concentration). The LRVs were found to be comprised between 0.47 and 1 log after the elution step where no significant difference was detected from the feed solution (p-value = 0.172). After the concentration step, all forest sites showed reductions in ΦX174 between 0.68 and 0.89 log, while ΦX174 spiked in grassland soils were reduced by between 1.07 and 1.34 log. Overall, the LRVs obtained after concentration step were found significant different from the feed solution (p-value = 0.02). Hueschterbach, Pall 1 and Pall 2 showed high LRVs after the concentration step, however, as the final quantity of ΦX174 remained satisfactory for these

three sites and the methodology was desired to be applied similarly to all sites, the results were considered satisfactory.

Table S6: Recovery of bacteriophage ΦX174 at each step of the elution and concentration protocol for each study site. Kruskal-Wallis one-way ANOVA on ranks was performed on the raw data at the level of confidence of 95%.

	Feed solution	Elution step		Concentration step	
Sites	ΦX174 quantity Mean ± sd (PFU)	ΦX174 quantity Mean ± sd (PFU)	LRV Mean ± sd	ΦX174 quantity Mean ± sd (PFU)	LRV Mean ± sd
Daerent	$2.04 \times 10^{9} \pm 2.83 \times 10^{7}$	$3.95 \times 10^{8} \pm 1.92 \times 10^{8}$	- 0.76 ± 0.27	$5.73 \times 10^{8} \pm 4.03 \times 10^{8}$	- 0.68 ± 0.47
Hueschterbach	$1.45 \times 10^{9} \pm 3.54 \times 10^{7}$	$1.29 \times 10^{8} \pm 4.04 \times 10^{6}$	- 1.05 ± 0.01	$6.65 \times 10^{7} \pm 1.06 \times 10^{7}$	- 1.34 ± 0.07
Koulbich	$1.84 \times 10^{9} \pm 1.41 \times 10^{7}$	$2.26 \times 10^{8} \pm 1.44 \times 10^{7}$	- 0.91 ± 0.03	$1.59 \times 10^{8} \pm 2.84 \times 10^{7}$	- 1.07 ± 0.07
Mollbach	$1.84 \times 10^{9} \pm 1.41 \times 10^{7}$	$1.70 \times 10^{8} \pm 7.55 \times 10^{6}$	- 1.03 ± 0.02	$1.59 \times 10^{8} \pm 2.60 \times 10^{7}$	- 1.07 ± 0.07
Pall 1	$1.72 \times 10^{9} \pm 7.07 \times 10^{6}$	$1.91 \times 10^{8} \pm 3.31 \times 10^{7}$	- 0.96 ± 0.08	$9.79 \times 10^{7} \pm 6.06 \times 10^{6}$	- 1.24 ± 0.03
Pall 2	$1.72 \times 10^{9} \pm 7.07 \times 10^{6}$	$2.06 \times 10^{8} \pm 3.20 \times 10^{7}$	- 0.92 ± 0.06	$1.23 \times 10^{8} \pm 3.66 \times 10^{7}$	- 1.14 ± 0.13
Retgenbusch	$2.04 \times 10^{9} \pm 2.83 \times 10^{7}$	$4.07 \times 10^{8} \pm 1.33 \times 10^{8}$	- 0.72 ± 0.15	$3.35 \times 10^{8} \pm 2.35 \times 10^{7}$	- 0.76 ± 0.03
Weierbach	$2.35 \times 10^{9} \pm 2.12 \times 10^{8}$	$7.97 \times 10^{8} \pm 1.00 \times 10^{8}$	- 0.47 ± 0.06	$3.56 \times 10^{8} \pm 2.03 \times 10^{8}$	- 0.89 ± 0.33

sd = standard deviation; LRV = Log reduction value; PFU = Plaque forming unit

Conclusion

The protocol for viral elution and concentration was optimised on three steps: mechanical detachment, chemical detachment and virus concentration. As a result, the mechanical detachment did not allow increasing the detachment rate of bacteriophage ΦX174. Therefore, only the chemical detachment using a solution mixing beef extract (3%) with glycine (0.05M) at pH 9.5 was performed followed by the concentration step using an Amicon 50 kDa. Then, all final solutions underwent a VLPs counting (See manuscript content) as well as DNA extraction (See supplementary material part B).

Section S2: Viral and total DNA extraction optimisation

Materials and method

As for the optimisation of the viral elution and concentration, the DNA extraction and library preparation were first conducted for the Weierbach, on both viral concentrate and soil sample, all in triplicate. Regarding the viral concentrates, a DNase treatment using Invitrogen™ kit TURBO DNA-free™ (AM1907, Thermo Fisher Scientific) was tested prior to DNA extraction to remove free DNA. Then, three different extraction kits were selected: the DNeasy® PowerSoil® kit (Qiagen, Hilden, Germany), QIAmp DNA mini kit (51304, Qiagen) and AllPrep PowerViral DNA/RNA kit (28000-50, Qiagen). The obtained DNA solutions were quantified using Invitrogen™ Qubit™ 2.0 fluorometer (ThermoFischer). Regarding the protocol on soil samples, only DNeasy® PowerSoil® kit and PowerViral kit were used as they were specifically conceived for soil sample extraction. On the extracted DNA from all extractions, library preparations were performed using Nextera XT DNA kit (FC-131-1096, Illumina) and the amplified DNA were quantified using qPCR (Applied biosystems ViiA 7, ThermoFisher). It is noteworthy that when the DNA quantity was detected below the limit detection of the device, the quantity was taken as recommended by the manufacturer of the library preparation protocol. Finally, paired-end sequence reads were generated using the Illumina MiSeq (2x350 bp). The quality control on the raw sequences was assessed using FastQC 0.11.8.

Results

The results obtained on the viral concentrates and soil samples are reported in Table S7. The viral concentrates showed better DNA recovery with the QiAmp kit than with the PowerSoil or PowerViral kits, for which the quantities were detected below the detection limit. However, the library preparations were not successful on the DNA extracted by QiAmp. Instead, despite the inability to quantify, libraries could be constructed on DNA extracts from PowerSoil and PowerViral kits. Nevertheless, the quantities of libraries were found to be lower than required (< 10^4 pM), making sequencing impossible. The DNA extracted with the PowerSoil kit on the soil samples were, in contrary, sufficient to successfully construct the libraries (> 10^4 pM), which was not observed for the extraction performed with PowerViral. The libraries from PowerSoil kit were

sequenced using MiSeq sequencer, but the reads produced was revealed to have very poor quality (Figure S2).

A

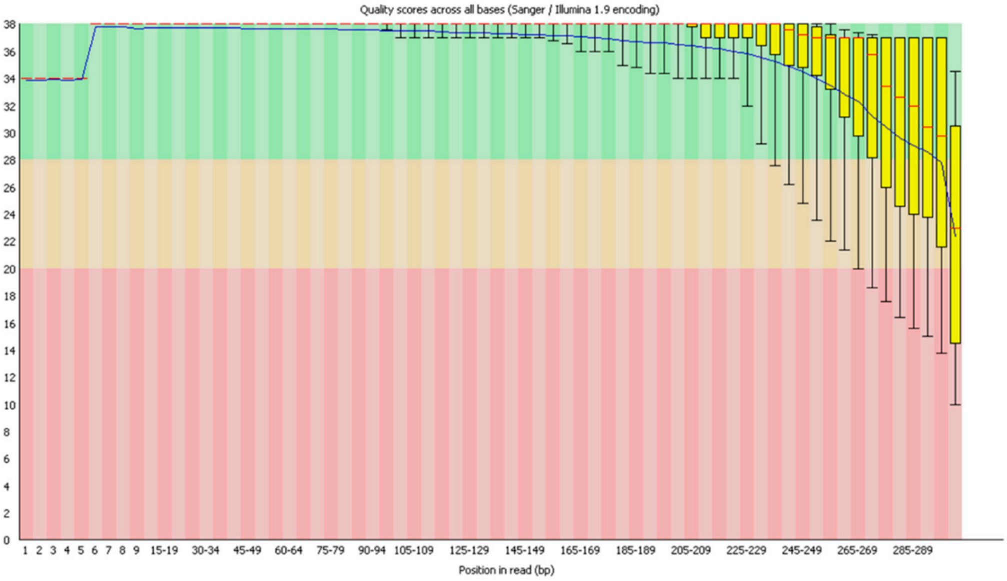

B

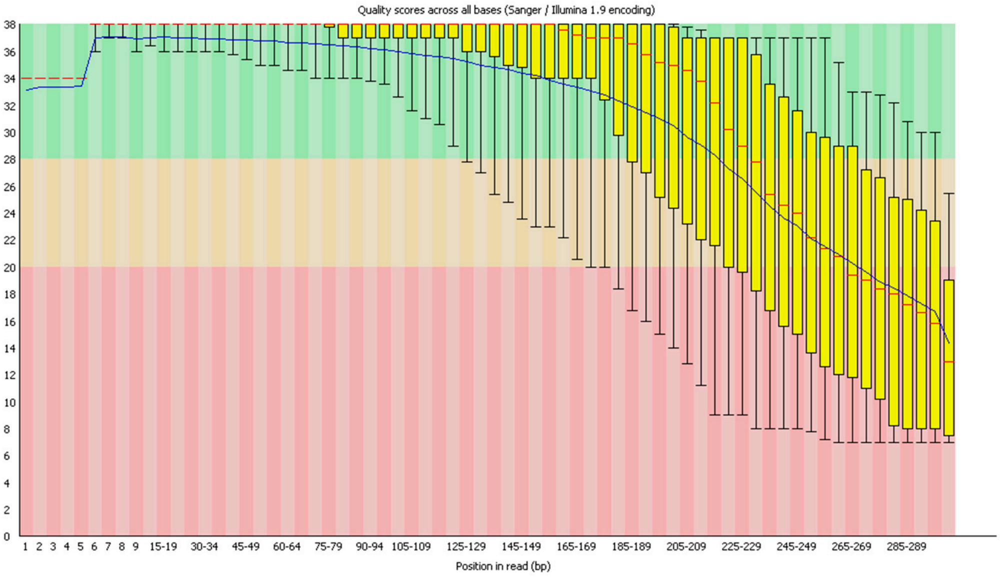

Figure S2: Per base sequence quality from FastQC for read forward 1 (A) and read reverse 2 (B) of the sequencing ot the Weierbach soil.

Table S7: Summary of each step from the DNase treatment to the library preparation from viral concentrates and soil sample of the Weierbach site.

	Viral concentrate					Soil sample	
**Initial volume/mass**	200 μL	200 μL	200 μL	200 μL	200 μL	250 μg	250 μg
**DNase treatment**	Yes	No	Yes	No	Yes	No	No
**DNA extraction kit**	PowerSoil	PowerSoil	QIAmp	QIAmp	PowerViral	PowerSoil	PowerViral
**DNA quantification (mean)**	< 0.05 ng/μL*	< 0.05 ng/μL*	0.575 ng/μL [1]	0.551 ng/μL [1]	< 0.05 ng/μL*	51 ng/μL	25.5 ng/μL
**Libraries quantification (mean)**	0.8 pM	2 pM	< DL	< DL	25.5 pM	2.74×10^4 pM	2.74×10^3 pM

* Below the detection limit of the Qubit 2.0; DL = Detection Limit.

[1] It is noteworthy that a cleaning step using DNeasy PowerClean CleanUp kit (12877-50, Qiagen) was tested on the extracted DNA obtained through the QIAmp kit to increase the DNA concentrations. However, the obtained concentrations were detected below the detection limit of the Qubit 2.0, thus the remaining steps were not carried out.

Conclusion

The DNA extractions followed by the library preparations were not successfully addressed to run the sequencing on the viral concentrates. On the contrary, the libraries obtained from the soil samples were sequenced but revealed very poor quality. Therefore, future analyses were explored, particularly the use of Nextera Flex kit followed by NovaSeq 6000 sequencer, which resulted in raw sequences from the soil samples, as described in the main manuscript.

References

97. Chen, L.; Xun, W.; Sun, L.; Zhang, N.; Shen, Q.; Zhang, R. Effect of Different Long-Term Fertilization Regimes on the Viral Community in an Agricultural Soil of Southern China. *Eur. J. Soil Biol.* **2014**, *62*, 121–126, doi:10.1016/j.ejsobi.2014.03.006.
98. Doan, T.T.; Bouvier, C.; Bettarel, Y.; Bouvier, T.; Henry-des-Tureaux, T.; Janeau, J.L.; Lamballe, P.; Nguyen, B. Van; Jouquet, P. Influence of Buffalo Manure, Compost, Vermicompost and Biochar Amendments on Bacterial and Viral Communities in Soil and Adjacent Aquatic Systems. *Appl. Soil Ecol.* **2014**, *73*, 78–86, doi:10.1016/j.apsoil.2013.08.016.
99. Williamson, K.E.; Corzo, K.A.; Drissi, C.L.; Buckingham, J.M.; Thompson, C.P.; Helton, R.R. Estimates of Viral Abundance in Soils Are Strongly Influenced by Extraction and Enumeration Methods. *Biol. Fertil. Soils* **2013**, *49*, 857–869, doi:10.1007/s00374-013-0780-z.
100. Williamson, K.E.; Wommack, K.E.; Radosevich, M. Sampling Natural Viral Communities from Soil for Culture-Independent Analyses. *Appl. Environ. Microbiol.* **2003**, *69*, 6628–6633, doi:10.1128/AEM.69.11.6628-6633.2003.
101. Swanson, M.M.; Fraser, G.; Daniell, T.J.; Torrance, L.; Gregory, P.J.; Taliansky, M. Viruses in Soils: Morphological Diversity and Abundance in the Rhizosphere. *Ann. Appl. Biol.* **2009**, *155*, 51–60, doi:10.1111/j.1744-7348.2009.00319.x.
102. Ateba, C.N.; Akindolire, M.A. Isolation and Characterisation of Bacteriophages with Lytic Activity Against Virulent Escherichia Coli O157:H7: Potential Bio-Control Agents. **2019**, doi:10.20944/PREPRINTS201901.0132.V1.
103. Gupta, V.; Saxena, H.M. Isolation and Characterization of BpL1, a Broad Acting Lytic Bacteriophage against Brucella. *Int. J. Curr. Microbiol. Appl. Sci.* **2017**, *6*, 2486–2496, doi:10.20546/IJCMAS.2017.611.292.
104. Hyman, P. Phages for Phage Therapy: Isolation, Characterization, and Host Range Breadth. *Pharmaceuticals* **2019**, *12*, doi:10.3390/PH12010035.
