# Supplementary material for: Soil pH, Calcium Content and Bacteria as Major Factors Responsible for the Distribution of the Known Fraction of the DNA Bacteriophage Populations in Soils of Luxembourg
Source: Microorganisms. 2022 Jul 19;10(7):1458. doi: 10.3390/microorganisms10071458 (PMC9321959; doi:10.3390/microorganisms10071458)
Supplement: Supplementary file 1 [file microorganisms-10-01458-s001.zip › Supplementary_TableS1_v1.pdf]

Supplementary Table S1: Percentages of relative abundances identified for each taxonomic domain

Sites	Bacteria	Eukaryota	Archaea	Viruses	Unclassified
Daerent	74.49%	0.30%	0.21%	0.030%	24.97%
Weierbach	75.51%	0.32%	0.33%	0.011%	23.37%
Retgenbusch	75.51%	0.48%	0.13%	0.016%	23.85%
Mollbach	75.04%	0.39%	0.11%	0.020%	24.43%
Koulbich	75.21%	0.43%	0.22%	0.014%	24.12%
Hueschterbach	76.36%	0.22%	0.21%	0.020%	23.19%
Pall1	76.16%	0.17%	0.20%	0.025%	23.44%
Pall 2	75.00%	0.35%	0.18%	0.020%	24.45%
