# Supplementary material for: Soil pH, Calcium Content and Bacteria as Major Factors Responsible for the Distribution of the Known Fraction of the DNA Bacteriophage Populations in Soils of Luxembourg
Source: Microorganisms. 2022 Jul 19;10(7):1458. doi: 10.3390/microorganisms10071458 (PMC9321959; doi:10.3390/microorganisms10071458)
Supplement: Supplementary file 1 [file microorganisms-10-01458-s001.zip › Supplementary_TableS2_v1.pdf]

microorganisms

Supplementary Table S2: Shannon diversity index (H), evenness (E) and species richness (S) of bacteria populations in the soil of the studied sites

Sites	Shannon Index (H)	Evenness (E)	Species Richness (S)
Daerent	3.12	0.320	17,016
Hueschterbach	3.11	0.320	16,799
Koulbich	3.13	0.317	19,063
Mollbach	3.11	0.317	18,165
Pall 1	3.18	0.325	17,381
Pall 2	3.13	0.320	17,943
Retgenbusch	2.78	0.290	15,247
Weierbach	2.89	0.30	15,272
